# Immune function genes *CD99L2, JARID2* and *TPO* show association with autism spectrum disorder

**DOI:** 10.1186/2040-2392-3-4

**Published:** 2012-06-09

**Authors:** Paula S Ramos, Satria Sajuthi, Carl D Langefeld, Stephen J Walker

**Affiliations:** 1 Division of Rheumatology and Immunology, Department of Medicine, Medical University of South Carolina, 96 Jonathan Lucas St., Suite 912, Charleston, SC 29425, USA; 2 Department of Biostatistical Sciences and Center for Public Health Genomics, Division of Public Health Sciences, Wake Forest School of Medicine, Medical Center Boulevard, WC 23, Winston Salem, NC, 27157, USA; 3 Wake Forest Institute for Regenerative Medicine, Wake Forest School of Medicine, 391 Technology Way, Winston-Salem, NC, 27101, USA

**Keywords:** Autism Spectrum Disorders, immune loci, family-based association analysis, single-nucleotide polymorphism

## Abstract

****Background**:**

A growing number of clinical and basic research studies have implicated immunological abnormalities as being associated with and potentially responsible for the cognitive and behavioral deficits seen in autism spectrum disorder (ASD) children. Here we test the hypothesis that immune-related gene loci are associated with ASD.

****Findings**:**

We identified 2,012 genes of known immune-function via Ingenuity Pathway Analysis. Family-based tests of association were computed on the 22,904 single nucleotide polymorphisms (SNPs) from the 2,012 immune-related genes on 1,510 trios available at the Autism Genetic Resource Exchange (AGRE) repository. Several SNPs in immune-related genes remained statistically significantly associated with ASD after adjusting for multiple comparisons. Specifically, we observed significant associations in the CD99 molecule-like 2 region (*CD99L2*, rs11796490, *P* = 4.01 × 10^-06^, OR = 0.68 (0.58-0.80)), in the jumonji AT rich interactive domain 2 (*JARID2*) gene (rs13193457, *P* = 2.71 × 10^-06^, OR = 0.61 (0.49-0.75)), and in the thyroid peroxidase gene (*TPO*) (rs1514687, *P* = 5.72 × 10^-06^, OR = 1.46 (1.24-1.72)).

****Conclusions**:**

This study suggests that despite the lack of a general enrichment of SNPs in immune function genes in ASD children, several novel genes with known immune functions are associated with ASD.

## **Findings**

### **Background**

Autism spectrum disorders (ASDs) encompass a heterogeneous group of clinical descriptors whose core features include deficits in cognition, communication and social acuity, coupled with stereotypical behaviors. Currently, 1 in 88 children in the United States have an ASD diagnosis, with boys being affected at a ratio of 5:1 compared to girls [1]. The etiology of ASDs, with very few exceptions, is unknown although a growing number of clinical and basic research studies have implicated immunological abnormalities as being associated with and potentially responsible for the cognitive and behavioral deficits seen in ASD children [2-4].

A judicious and efficient way to identify genetic variation predisposing to complex diseases is the application of a hypothesis-driven framework that incorporates prior biological knowledge. Unlike agnostic genome wide association studies (GWAS), this approach narrows the hypothesis space to provide a more focused and powerful examination of the data. Indeed, several studies have shown that a hypothesis-driven approach of selecting candidate genes serves to increase the reliability and likelihood of finding genes that are truly associated with disease [5,6]. In accord with these considerations, we tested for associations between immune-related genes and ASD children.

### **Approach**

We used Ingenuity Pathway Analysis (IPA) [7], a bioinformatics database, to search for molecules with known immune function. IPA offers the largest and most comprehensive set of functional annotations, integrating manually curated data from other databases, and a broad range of scientific literature. Briefly, we searched for “immune” under Functions and Diseases, exported the molecule annotations, and used MatchMiner [8] to get the positions of the corresponding 2,012 immune-function genes (Additional file 1: Table S1).

We then used the genotypic data available at the Autism Genetic Resource Exchange (AGRE) [9] repository to perform a family-based association analysis between variants in those 2,012 immune loci and ASD. These data have previously been used in several GWAS [10-12], and the families used in our analysis are, with the exception of minor differences due to slightly different quality control criteria, the same ones as reported in Ma *et al.*[10], Wang *et al.*[11] and Weiss *et al.*[12]. These data and families also overlap with those reported by Voineagu *et al.*[13], who, included in their expression profiling study, used the AGRE data to perform an analysis searching for evidence of a genetic enrichment of immune loci. We searched for SNPs in a region including 5 kb up- and downstream of each immune gene. After applying quality control filters, we identified 22,904 SNPs genotyped in the AGRE collection of 1,510 trios on the Illumina Hap550 platform. We tested these SNPs for association with ASD using the standard Transmission Disequilibrium Test (TDT), as implemented in PLINK v1.07 [14]. In addition, given that the 1,510 trios are part of 1,057 independent nuclear families, we also used the Pedigree Disequilibrium Test (PDT), which uses data from related nuclear families and discordant sibships from extended pedigrees [15]. The following exclusion filters were applied: minor allele frequency (MAF) < 0.01, SNP genotyping missing rate > 10%, individuals > 10% missing genotypes, Hardy-Weinberg Equilibrium (HWE) *P*-value < 0.001 (founders), Mendelian error rate > 5% per family and > 4% per SNP.

Since we hypothesized that ASD children may be enriched for risk loci in immune genes, we tested for a potential enrichment of significant *P*-values for SNPs in immune-related genes. We tested for an enrichment of association signals in these genes using INRICH [16], which corrects the empirical gene-set *P*-value using Bootstrapping-based re-sampling. In addition, we assessed the distribution of the linkage-disequilibrium (LD)-pruned immune-gene-set with random LD-pruned SNPs, using an r^2^ > 0.2 for LD-based SNP pruning. We did not observe an enrichment of immune-gene associations with either test (*P* = 1.0 and *P* = 0.45, respectively).

In order to adjust for multiple testing we applied a Benjamini and Hochberg false discovery rate (FDR) correction [17] to the 22,904 SNPs analyzed (P-FDR). Only three SNPs remained statistically significant (P-FDR < 0.05) (Table 1). Under an additive model, and at alpha = 1.0E-05, this sample has >80% power to detect the associations reported in Table 1.

**Table 1:** Most significant immune loci from the association analyses

**SNP**	**Chr**	**Pos (Mb)**	**Region**	**TDT**					**PDT**
				**MA**	**T:U**	**OR (95% CI)**	***P*-value**	**P-FDR**	**P-value**
rs7880807^I^	X	15.006	CD99L2	A	190:299	0.64 (0.53-0.76)	8.26E-07	-	ND
rs13193457*	6	15.346	JARID2	A	139:229	0.61 (0.49-0.75)	2.71E-06	-	6.04E-05
rs11796490	X	150.058	CD99L2	G	239:351	0.68 (0.58-0.80)	4.01E-06	1.53E-02	NA
rs1514687	2	1.438	TPO	A	349:239	1.46 (1.24-1.72)	5.72E-06	1.87E-02	4.49E-06
rs10890518^I^	2	1.435	TPO	G	356:249	1.43 (1.22-1.62)	1.36E-05	-	ND

Chr, Chromosome; Pos., position; MA, minor allele; T:U, transmitted to untransmitted minor allele counts; OR, odds ratio; CI, confidence interval; P-FDR. False Discovery Rate-adjusted *P*-value; ND, test not done with imputed data; NA, not applicable, as the PDT does not handle chromosome X data. *This SNP had a 5% Mendelian error rate. ^I^This SNP was imputed.

Finally, in order to fine map the novel loci, we used BEAGLE v3.3.2 [18] and the 1000 Genomes Project Phase 1 (version 3; http://www.1000genomes.org/) reference panel to impute all SNPs in the regions of the top genes.

### **Novel immune genes**

The lack of an enrichment of significant associations in immune genes corroborates the findings by Voineagu *et al.*[13], who used a complimentary approach to test for evidence of a genetic component for the up-regulation of immune response genes in the autistic brain using the same AGRE genotype data. Nevertheless, we uncovered a few associations in immune-related genes that met an FDR-adjustment.

**Figure 1:**
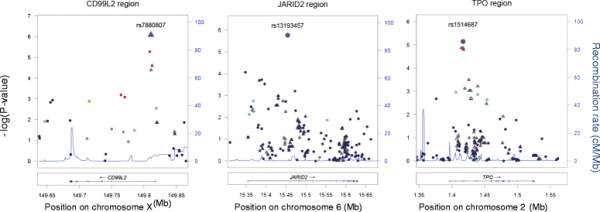
**Regional plots of immune ASD loci.** Genotyped (circles) and imputed (triangles) SNPs are plotted with their *P*-values (as -log_10_ values) as a function of genomic position (Human Genome Build 18) within a region surrounding the most significant SNP (purple color). Recombination rates from the HapMap Phase II CEU are plotted in blue to reflect the regional LD structure. In each region, the index SNP is represented by a large purple symbol, and the color of all other SNPs indicates LD with the index SNP based on pairwise *r*^2^ values from HapMap CEU (red, *r*^2^ > 0.8; orange, *r*^2^ = 0.6 to 0.8; green, *r*^2^ = 0.4 to 0.6; light blue, *r*^2^ = 0.2 to 0.4; dark blue, *r*^2^ < 0.2). Known human genes in the UCSC Genome Browser are in the bottom.

We observed a significant association in the CD99 molecule-like 2 gene (*CD99L2*) (rs11796490, *P* = 4.01x10^-06^, OR = 0.68 (0.58-0.80)) (Table 1). An imputed variant in LD with this genotyped SNP showed even stronger association (rs7880807, *P* = 8.26 x 10^-07^, OR = 0.64 (0.53-0.76)) (r^2^ = 1.0 in CEU from HapMap release 21) (Figure 1). As is evident from Figure 1, other variants in this gene are also showing evidence of association with ASD. Although more modest, these associations with neighboring SNPs corroborate the association observed at rs11796490, which is, therefore, less likely to be spurious. This variant is located in the first intron of *CD99L2*. The product of this gene functions as an adhesion molecule during leukocyte extravasation, in particular at the diapedesis step.

A variant in the first intron of the jumonji AT rich interactive domain 2 (*JARID2*) gene showed association with ASD (rs13193457, *P* = 2.71 × 10^-06^, OR = 0.61 (0.49-0.75) (Table 1). Despite having a borderline Mendelian error rate, this SNP is of interest. This gene has been previously implicated in schizophrenia [19,20] and, more recently, it has been reported in a GWAS for ASD that also included the same AGRE dataset herein used [12]. The variant reported by Weiss *et al.* (rs7766973) does not show LD with rs13193457 (r^2^ = 0.13 in CEU from 1000 Genomes Pilot 1). The jumonji-domain functions by removing methyl marks on histones that are associated with gene regulation [21]. JARID2 is required for neural tube formation and is essential for normal heart development and function, as well as acting as a transcriptional repressor of ANF by binding to both GATA4 and NKX2-5 and repressing their transcriptional activator activities.

An intronic variant in the thyroid peroxidase gene (*TPO*) has also shown association with ASD (rs1514687, *P* = 5.72 × 10^-06^, OR = 1.46 (1.24-1.72)). This association is corroborated by those of neighboring SNPs (Figure 1). This gene has been associated with hypothyroidism [22]. This gene encodes a membrane-bound glycoprotein which acts as an enzyme and plays a central role in thyroid gland function. The protein functions in the iodination of tyrosine residues in thyroglobulin and phenoxy-ester formation between pairs of iodinated tyrosines to generate the thyroid hormones, thyroxine and triiodothyronine. Mutations in this gene are associated with several disorders of thyroid hormonogenesis, including congenital hypothyroidism, congenital goiter and thyroid hormone organification defect IIA. Multiple transcript variants encoding distinct isoforms have been identified for this gene.

## **Discussion**

In spite of its reported high heritability, the identification of genetic risk factors predisposing to ASDs has been difficult. To date, only a small number of loci are considered established (that is, have met genome-wide significance and have been replicated in independent cohorts). GWAS are by design agnostic. Increased statistical power can be achieved with more focused hypotheses. Given the increasing speculation of a significant role for immune genes in the etiology and pathogenesis of ASD, we undertook a search for genetic variation associated with ASD in genes with immune functions. This hypothesis-driven candidate gene approach based on prior knowledge was selected because it serves to increase the power, reliability and likelihood of finding genes that are associated with disease [5,6].

A lack of enrichment of genetic associations in immune-related genes has recently been reported [13] in the same dataset we used, and this conclusion is supported here using another statistical approach. The associations we uncovered in immune-related genes and the observed dysregulation of immune genes in ASD [13] suggest that variation in a limited number of immune-function genes may be responsible for observed up-regulation of their immune downstream targets.

The transmission/disequilibrium test (TDT) is largely robust to population substructure as it is a within-family comparison. The individuals in these data are predominately of European descent (76%), with Asian (3%) and African (2%) ancestry represented; the Autism Genetic Resource Exchange (AGRE) repository lists 14% and 5% of the individuals of unknown or admixed ancestry. One limitation of using the TDT in our study is the lack of total independence between the trios analyzed: the 1,510 trios used in the TDT are part of 1,101 independent nuclear families. The correlation among children in the same family can inflate the type I error probability. We, thus, also applied the PDT, also a within family test that is robust to population substructure, and the PDT analysis corroborates the TDT analysis.

## **Conclusion**

SNPs within the CD99 molecule-like 2 gene, the jumonji AT rich interactive domain 2 (*JARID2*) gene, and the thyroid peroxidase gene were associated with ASD after multiple comparisons adjustment. Understanding how these genetic factors might contribute to pathogenesis should ultimately lead to important opportunities for discovering therapeutic targets that can be used to treat ASD.

## **Abbreviations**

AGRE: Autism Genetic Resource Exchange; ASDs: Autism Spectrum Disorders; CI: confidence interval; FDR: False Discovery Rate; GWAS: genome wide association studies; HWE: Hardy-Weinberg Equilibrium; IPA: Ingenuity Pathway Analysis; JARID2: jumonji AT rich interactive domain 2; MAF: minor allele frequency; OR: odds ratio; PDT: Pedigree Disequilibrium Test; P-FDR: False Discovery Rate-adjusted P-value; SNP: single nucleotide polymorphism; TDT: Transmission Disequilibrium Test; TPO: thyroid peroxidase gene.

## **Competing interests**

The authors declare that they have no competing interests.

## **Authors' contributions**

PSR conceived the study, participated in the design of the study, performed statistical analysis and results interpretation, and drafted the manuscript. SS performed statistical analysis. CDL conceived the study, participated in the design of the study, coordinated the study and drafted the manuscript. SJW conceived the study, participated in its design and drafted the manuscript. All authors read and approved the final manuscript.

## Supplementary Material

Additional file 1: **Table S1.** Immune-function genes. Click here for file
