# Supplementary material for: Immune function genes CD99L2, JARID2 and TPO show association with autism spectrum disorder
Source: Mol Autism. 2012 Jun 9;3:4. doi: 10.1186/2040-2392-3-4 (PMC3439713; doi:10.1186/2040-2392-3-4)
Supplement: Additional file 1: — Table S1. Immune-function genes. [file 2040-2392-3-4-S1.doc]

Supplemental Table 1. Immune-function genes.

| Chromosome | Start bp | End bp | Gene symbol |
| --- | --- | --- | --- |
| 1 | 938741 | 939780 | ISG15 |
| 1 | 1128751 | 1131952 | TNFRSF18 |
| 1 | 1136569 | 1139375 | TNFRSF4 |
| 1 | 1971768 | 2106692 | PRKCZ |
| 1 | 2479152 | 2486613 | TNFRSF14 |
| 1 | 2975603 | 3345043 | PRDM16 |
| 1 | 3558988 | 3639716 | TP73 |
| 1 | 6443807 | 6448842 | TNFRSF25 |
| 1 | 7830261 | 7836159 | UTS2 |
| 1 | 7902494 | 7923474 | TNFRSF9 |
| 1 | 9634389 | 9711556 | PIK3CD |
| 1 | 10432557 | 10434544 | CORT |
| 1 | 10443191 | 10455200 | DFFA |
| 1 | 11089179 | 11245151 | MTOR |
| 1 | 11828362 | 11830422 | NPPA |
| 1 | 11962955 | 11996152 | MFN2 |
| 1 | 12046020 | 12126849 | TNFRSF8 |
| 1 | 12149646 | 12191863 | TNFRSF1B |
| 1 | 15691383 | 15723377 | CASP9 |
| 1 | 16323419 | 16355151 | EPHA2 |
| 1 | 19842312 | 19857532 | NBL1 |
| 1 | 20119387 | 20122697 | PLA2G2E |
| 1 | 20174517 | 20179496 | PLA2G2A |
| 1 | 20269287 | 20290980 | PLA2G5 |
| 1 | 20311027 | 20318595 | PLA2G2D |
| 1 | 20788030 | 20817985 | CDA |
| 1 | 20850846 | 20860624 | DDOST |
| 1 | 21795301 | 21868386 | RAP1GAP |
| 1 | 21877380 | 21982275 | IMP |
| 1 | 22251706 | 22292022 | CDC42 |
| 1 | 22318786 | 22342197 | WNT4 |
| 1 | 22835704 | 22838761 | C1QA |
| 1 | 22842733 | 22847189 | C1QC |
| 1 | 22852268 | 22860615 | C1QB |
| 1 | 23705508 | 23730300 | E2F2 |
| 1 | 23757042 | 23758872 | ID3 |
| 1 | 23990232 | 23994614 | LYPLA2 |
| 1 | 24073047 | 24112404 | CNR2 |
| 1 | 24353234 | 24386338 | IL28RA |
| 1 | 25098589 | 25164088 | RUNX3 |
| 1 | 27110565 | 27113047 | NR0B2 |
| 1 | 27811388 | 27834314 | FGR |
| 1 | 27865158 | 27871311 | IFI6 |
| 1 | 28348450 | 28375778 | PTAFR |
| 1 | 28935722 | 28968873 | YTHDF2 |
| 1 | 29011240 | 29062795 | OPRD1 |
| 1 | 32417931 | 32436472 | TXLNA |
| 1 | 32489426 | 32524352 | LCK |
| 1 | 33013426 | 33056220 | YARS |
| 1 | 35031185 | 35033933 | GJA4 |
| 1 | 36704231 | 36721096 | CSF3R |
| 1 | 41717035 | 41722884 | EDN2 |
| 1 | 43576061 | 43592721 | MPL |
| 1 | 46632578 | 46652099 | FAAH |
| 1 | 47454550 | 47468030 | TAL1 |
| 1 | 47674275 | 47678950 | FOXD2 |
| 1 | 51206954 | 51212895 | CDKN2C |
| 1 | 52661537 | 52791331 | ZCCHC11 |
| 1 | 59019052 | 59022373 | JUN |
| 1 | 65071494 | 65204775 | JAK1 |
| 1 | 65658905 | 65875408 | LEPR |
| 1 | 66030780 | 66612849 | PDE4B |
| 1 | 67545634 | 67635170 | IL12RB2 |
| 1 | 67923470 | 67926607 | GADD45A |
| 1 | 71090623 | 71286079 | PTGER3 |
| 1 | 78858736 | 78880657 | IFI44L |
| 1 | 78888103 | 78902345 | IFI44 |
| 1 | 85504049 | 85516171 | BCL10 |
| 1 | 89345897 | 89364387 | GBP2 |
| 1 | 91920574 | 92124243 | TGFBR3 |
| 1 | 92484544 | 92537154 | GLMN |
| 1 | 92712909 | 92725021 | GFI1 |
| 1 | 93799937 | 93919973 | BCAR3 |
| 1 | 94767460 | 94779903 | F3 |
| 1 | 100957884 | 100977187 | VCAM1 |
| 1 | 101475042 | 101479661 | S1PR1 |
| 1 | 107915304 | 108309068 | VAV3 |
| 1 | 109892823 | 109938498 | GNAI3 |
| 1 | 110254979 | 110273877 | CSF1 |
| 1 | 110683467 | 110690826 | RBM15 |
| 1 | 110745401 | 110752069 | HBXIP |
| 1 | 110795310 | 110801497 | PROK1 |
| 1 | 111015832 | 111019178 | KCNA3 |
| 1 | 111635006 | 111664708 | CHIA |
| 1 | 111827492 | 111908120 | ADORA3 |
| 1 | 111963927 | 112057623 | RAP1A |
| 1 | 114157962 | 114215857 | PTPN22 |
| 1 | 115630059 | 115682380 | NGF |
| 1 | 116858679 | 116915126 | CD58 |
| 1 | 117098624 | 117113373 | CD2 |
| 1 | 117487732 | 117555072 | VTCN1 |
| 1 | 120255700 | 120413799 | NOTCH2 |
| 1 | 144149938 | 144153880 | TXNIP |
| 1 | 144287344 | 144297903 | PIAS3 |
| 1 | 144407154 | 144426922 | CD160 |
| 1 | 148020911 | 148030697 | FCGR1A |
| 1 | 148305965 | 148384128 | VPS45 |
| 1 | 148813660 | 148818760 | MCL1 |
| 1 | 148969177 | 149004929 | CTSS |
| 1 | 149493820 | 149506576 | PSMD4 |
| 1 | 150045172 | 150070972 | RORC |
| 1 | 150222010 | 150233338 | S100A10 |
| 1 | 151536961 | 151549818 | PGLYRP3 |
| 1 | 151569222 | 151587646 | PGLYRP4 |
| 1 | 151596953 | 151600126 | S100A9 |
| 1 | 151612807 | 151614699 | S100A12 |
| 1 | 151629132 | 151630173 | S100A8 |
| 1 | 151655623 | 151662325 | S100A7A |
| 1 | 151696844 | 151699761 | S100A7 |
| 1 | 152644292 | 152706812 | IL6R |
| 1 | 152806880 | 152818975 | CHRNB2 |
| 1 | 152821158 | 152867061 | ADAR |
| 1 | 153201398 | 153213464 | SHC1 |
| 1 | 153253547 | 153256076 | ZBTB7B |
| 1 | 153366972 | 153374008 | EFNA1 |
| 1 | 153424925 | 153429324 | MUC1 |
| 1 | 153470867 | 153481112 | GBA |
| 1 | 153526253 | 153537835 | PKLR |
| 1 | 155042659 | 155053226 | SH2D2A |
| 1 | 155052165 | 155118266 | NTRK1 |
| 1 | 156067329 | 156078175 | CD5L |
| 1 | 156416360 | 156422838 | CD1D |
| 1 | 156490550 | 156494680 | CD1A |
| 1 | 156526186 | 156531187 | CD1C |
| 1 | 156564365 | 156567945 | CD1B |
| 1 | 156590163 | 156593965 | CD1E |
| 1 | 157167965 | 157291567 | IFI16 |
| 1 | 157298898 | 157313271 | AIM2 |
| 1 | 157441133 | 157442912 | DARC |
| 1 | 157526127 | 157544637 | FCER1A |
| 1 | 157824239 | 157825284 | APCS |
| 1 | 157948703 | 157951003 | CRP |
| 1 | 158513627 | 158630410 | VIPR2 |
| 1 | 158721443 | 158759666 | SLAMF6 |
| 1 | 158782418 | 158815887 | CD84 |
| 1 | 158846513 | 158883493 | SLAMF1 |
| 1 | 158915161 | 158948209 | CD48 |
| 1 | 158975700 | 158991224 | SLAMF7 |
| 1 | 159066573 | 159099269 | CD244 |
| 1 | 159112955 | 159121584 | ITLN1 |
| 1 | 159232607 | 159275358 | F11R |
| 1 | 159275665 | 159282381 | USF1 |
| 1 | 159451710 | 159455662 | FCER1G |
| 1 | 159541150 | 159546377 | MPZ |
| 1 | 159741843 | 159755983 | FCGR2A |
| 1 | 159778174 | 159867782 | FCGR3A |
| 1 | 159859611 | 159867782 | FCGR3B |
| 1 | 159899563 | 159914575 | FCGR2B |
| 1 | 162795560 | 163082933 | PBX1 |
| 1 | 165456766 | 165651941 | POU2F1 |
| 1 | 165666507 | 165754450 | CD247 |
| 1 | 166812479 | 166817939 | XCL1 |
| 1 | 167824713 | 167866031 | SELP |
| 1 | 167926433 | 167947461 | SELL |
| 1 | 167958405 | 167969803 | SELE |
| 1 | 168157094 | 168310503 | KIFAP3 |
| 1 | 170894807 | 170902633 | FASLG |
| 1 | 171277073 | 171286679 | TNFSF18 |
| 1 | 171419493 | 171443094 | TNFSF4 |
| 1 | 171713108 | 171724570 | PRDX6 |
| 1 | 172139565 | 172153096 | SERPINC1 |
| 1 | 172172539 | 172228833 | RC3H1 |
| 1 | 177529639 | 177591075 | SOAT1 |
| 1 | 179269761 | 179291256 | MR1 |
| 1 | 180809394 | 180822731 | RNASEL |
| 1 | 181791320 | 181826339 | NCF2 |
| 1 | 183532146 | 183553084 | IVNS1ABP |
| 1 | 184907592 | 184916179 | PTGS2 |
| 1 | 185064654 | 185224728 | PLA2G4A |
| 1 | 190811479 | 190815782 | RGS1 |
| 1 | 191044793 | 191048026 | RGS2 |
| 1 | 194887763 | 195067939 | CFH |
| 1 | 195010552 | 195154385 | CFHR1 |
| 1 | 196874759 | 196993168 | PTPRC |
| 1 | 201176575 | 201194034 | ADIPOR1 |
| 1 | 201363458 | 201403156 | ADORA1 |
| 1 | 201452417 | 201465399 | CHIT1 |
| 1 | 201576375 | 201586912 | FMOD |
| 1 | 202000959 | 202012101 | LAX1 |
| 1 | 202390570 | 202402088 | REN |
| 1 | 204484081 | 204498726 | CTSE |
| 1 | 204710418 | 204736845 | IKBKE |
| 1 | 204747501 | 204829238 | RASSF5 |
| 1 | 204924911 | 204974249 | MAPKAPK2 |
| 1 | 205007570 | 205012462 | IL10 |
| 1 | 205038837 | 205082947 | IL19 |
| 1 | 205105776 | 205109190 | IL20 |
| 1 | 205168494 | 205186430 | PIGR |
| 1 | 205198132 | 205210117 | FCAMR |
| 1 | 205344229 | 205384931 | C4BPA |
| 1 | 205561487 | 205600469 | CD55 |
| 1 | 205694292 | 205729861 | CR2 |
| 1 | 205736095 | 205881732 | CR1 |
| 1 | 205992024 | 206035480 | CD46 |
| 1 | 206126506 | 206151306 | CD34 |
| 1 | 209566579 | 209614907 | TRAF5 |
| 1 | 216586490 | 216681593 | TGFB2 |
| 1 | 219941388 | 219982084 | DUSP10 |
| 1 | 221350206 | 221383247 | TLR5 |
| 1 | 224615128 | 224662414 | PARP1 |
| 1 | 224886014 | 224991818 | ITPKB |
| 1 | 226261374 | 226315584 | WNT3A |
| 1 | 228904891 | 228916564 | AGT |
| 1 | 233890970 | 234113563 | LYST |
| 1 | 234748187 | 234834437 | LGALS8 |
| 1 | 237858995 | 238139338 | CHRM3 |
| 1 | 240078157 | 240119670 | EXO1 |
| 2 | 1396241 | 1525502 | TPO |
| 2 | 8739563 | 8742032 | ID2 |
| 2 | 9546863 | 9613368 | ADAM17 |
| 2 | 11239228 | 11402162 | ROCK2 |
| 2 | 11804190 | 11884982 | LPIN1 |
| 2 | 15998133 | 16004579 | MYCN |
| 2 | 20264040 | 20288675 | SDC1 |
| 2 | 20510315 | 20512681 | RHOB |
| 2 | 20681044 | 20714345 | HS1BP3 |
| 2 | 21077806 | 21120450 | APOB |
| 2 | 24086456 | 24140052 | FKBP1B |
| 2 | 25237225 | 25245063 | POMC |
| 2 | 27383768 | 27384634 | UCN |
| 2 | 31410691 | 31491115 | XDH |
| 2 | 32303023 | 32344305 | NLRC4 |
| 2 | 33025895 | 33478077 | LTBP1 |
| 2 | 37187202 | 37229907 | EIF2AK2 |
| 2 | 45732546 | 46268632 | PRKCE |
| 2 | 46779602 | 46843430 | SOCS5 |
| 2 | 47483766 | 47563864 | MSH2 |
| 2 | 47863789 | 47887595 | MSH6 |
| 2 | 48767424 | 48836367 | LHCGR |
| 2 | 55368483 | 55500467 | CCDC88A |
| 2 | 60531806 | 60634137 | BCL11A |
| 2 | 60962255 | 61003682 | REL |
| 2 | 61558573 | 61618922 | XPO1 |
| 2 | 62276765 | 62305368 | B3GNT2 |
| 2 | 66516035 | 66653394 | MEIS1 |
| 2 | 69995706 | 70023578 | MXD1 |
| 2 | 70292319 | 70329251 | TIA1 |
| 2 | 70910855 | 70916461 | CD207 |
| 2 | 74635367 | 74638170 | DOK1 |
| 2 | 75129737 | 75279781 | TACR1 |
| 2 | 79106333 | 79109138 | REG3G |
| 2 | 85475381 | 85491187 | CAPG |
| 2 | 85774924 | 85779391 | GNLY |
| 2 | 86865239 | 86871638 | CD8A |
| 2 | 86895972 | 86942549 | CD8B |
| 2 | 88203624 | 88208693 | FABP1 |
| 2 | 95326798 | 95415551 | KCNIP3 |
| 2 | 96142352 | 96145615 | ADRA2B |
| 2 | 97696462 | 97722755 | ZAP70 |
| 2 | 101974737 | 102011312 | IL1R2 |
| 2 | 102136833 | 102162765 | IL1R1 |
| 2 | 102294393 | 102334929 | IL1RL1 |
| 2 | 102345528 | 102381647 | IL18R1 |
| 2 | 102401685 | 102435454 | IL18RAP |
| 2 | 105343716 | 105421392 | FHL2 |
| 2 | 105727785 | 105877158 | NCK2 |
| 2 | 111594961 | 111642493 | BCL2L11 |
| 2 | 112372661 | 112503416 | MERTK |
| 2 | 113247963 | 113259442 | IL1A |
| 2 | 113303807 | 113310827 | IL1B |
| 2 | 113479919 | 113482090 | IL1F6 |
| 2 | 113591940 | 113608063 | IL1RN |
| 2 | 119416214 | 119468707 | MARCO |
| 2 | 120726883 | 120768753 | RALB |
| 2 | 120819468 | 120825444 | INHBB |
| 2 | 127778608 | 127817240 | MAP3K2 |
| 2 | 127892486 | 127903286 | PROC |
| 2 | 134728299 | 134922938 | MGAT5 |
| 2 | 136261884 | 136311220 | LCT |
| 2 | 136588389 | 136592195 | CXCR4 |
| 2 | 151835230 | 151854620 | NMI |
| 2 | 151922350 | 151944805 | TNFAIP6 |
| 2 | 152402388 | 152663790 | CACNB4 |
| 2 | 152683352 | 152740752 | STAM2 |
| 2 | 157979377 | 158008850 | CYTIP |
| 2 | 158513627 | 158630410 | VIPR2 |
| 2 | 160368113 | 160469508 | LY75 |
| 2 | 160666479 | 160764836 | ITGB6 |
| 2 | 162557002 | 162639298 | DPP4 |
| 2 | 162831835 | 162883285 | IFIH1 |
| 2 | 166763053 | 166876560 | SCN9A |
| 2 | 171381445 | 171425903 | GAD1 |
| 2 | 173000559 | 173079427 | ITGA6 |
| 2 | 174481504 | 174538309 | SP3 |
| 2 | 175132548 | 175255873 | WIPF1 |
| 2 | 175320569 | 175337427 | CHRNA1 |
| 2 | 175372337 | 175578361 | CHN1 |
| 2 | 177803284 | 177837663 | NFE2L2 |
| 2 | 179004395 | 179024110 | PRKRA |
| 2 | 182029863 | 182110711 | ITGA4 |
| 2 | 187163044 | 187253872 | ITGAV |
| 2 | 187916093 | 188021266 | CALCRL |
| 2 | 188037204 | 188127464 | TFPI |
| 2 | 188865634 | 189168895 | GULP1 |
| 2 | 189547343 | 189585717 | COL3A1 |
| 2 | 191542121 | 191587181 | STAT1 |
| 2 | 191602550 | 191724170 | STAT4 |
| 2 | 196710102 | 196744520 | STK17B |
| 2 | 198059554 | 198073243 | HSPD1 |
| 2 | 198073364 | 198076416 | HSPE1 |
| 2 | 201158975 | 201244460 | AOX1 |
| 2 | 201689134 | 201737246 | CFLAR |
| 2 | 201806410 | 201860679 | CASP8 |
| 2 | 204279442 | 204310801 | CD28 |
| 2 | 204440753 | 204446928 | CTLA4 |
| 2 | 204509747 | 204534541 | ICOS |
| 2 | 208102930 | 208171806 | CREB1 |
| 2 | 213572657 | 213724578 | IKZF2 |
| 2 | 215933425 | 216009036 | FN1 |
| 2 | 216682376 | 216779248 | XRCC5 |
| 2 | 218698990 | 218710220 | IL8RB |
| 2 | 218735814 | 218739961 | IL8RA |
| 2 | 218954995 | 218969860 | SLC11A1 |
| 2 | 219648291 | 219733767 | NHEJ1 |
| 2 | 220145197 | 220148671 | INHA |
| 2 | 221990992 | 222145254 | EPHA4 |
| 2 | 224169903 | 224175365 | SCG2 |
| 2 | 227308181 | 227372719 | IRS1 |
| 2 | 227737524 | 227887751 | COL4A3 |
| 2 | 228386813 | 228390494 | CCL20 |
| 2 | 230798689 | 230886174 | SP140 |
| 2 | 231681198 | 231698068 | HTR2B |
| 2 | 232281479 | 232286493 | PTMA |
| 2 | 232951591 | 232955841 | ALPP |
| 2 | 233633279 | 233781287 | INPP5D |
| 2 | 237131746 | 237155731 | CXCR7 |
| 2 | 238060616 | 238128699 | MLPH |
| 2 | 239635318 | 239987580 | HDAC4 |
| 2 | 242440710 | 242449731 | PDCD1 |
| 3 | 3086422 | 3127031 | IL5RA |
| 3 | 4510135 | 4864079 | ITPR1 |
| 3 | 8750495 | 8763450 | CAV3 |
| 3 | 10181562 | 10260427 | IRAK2 |
| 3 | 10302433 | 10307409 | GHRL |
| 3 | 11269399 | 11279415 | HRH1 |
| 3 | 12304348 | 12450854 | PPARG |
| 3 | 12600107 | 12680678 | RAF1 |
| 3 | 15271360 | 15357905 | SH3BP5 |
| 3 | 16949585 | 17107089 | PLCL2 |
| 3 | 18364438 | 18440344 | SATB1 |
| 3 | 25444757 | 25614424 | RARB |
| 3 | 27732890 | 27738789 | EOMES |
| 3 | 30622997 | 30710637 | TGFBR2 |
| 3 | 32968069 | 32971407 | CCR4 |
| 3 | 37009982 | 37067339 | MLH1 |
| 3 | 38155008 | 38159514 | MYD88 |
| 3 | 38564556 | 38666167 | SCN5A |
| 3 | 39279989 | 39296531 | CX3CR1 |
| 3 | 39346218 | 39351077 | CCR8 |
| 3 | 41216015 | 41256938 | CTNNB1 |
| 3 | 42519120 | 42554063 | VIPR1 |
| 3 | 42825979 | 42883778 | CCBP2 |
| 3 | 43303099 | 43367634 | SNRK |
| 3 | 45903022 | 45919671 | CCR9 |
| 3 | 45959976 | 45964849 | CXCR6 |
| 3 | 46037295 | 46043983 | XCR1 |
| 3 | 46218203 | 46224836 | CCR1 |
| 3 | 46258691 | 46283166 | CCR3 |
| 3 | 46370363 | 46377427 | CCR2 |
| 3 | 46387429 | 46392699 | CCR5 |
| 3 | 46452500 | 46481399 | LTF |
| 3 | 47602390 | 47798410 | SMARCC1 |
| 3 | 48239865 | 48241979 | CAMP |
| 3 | 48420266 | 48441746 | PLXNB1 |
| 3 | 49036771 | 49041879 | IMPDH2 |
| 3 | 49369614 | 49370795 | GPX1 |
| 3 | 49371584 | 49424530 | RHOA |
| 3 | 49696392 | 49701099 | MST1 |
| 3 | 49899441 | 49916074 | MST1R |
| 3 | 50248650 | 50271790 | GNAI2 |
| 3 | 50618929 | 50624207 | CISH |
| 3 | 52230137 | 52235219 | TLR9 |
| 3 | 52504395 | 52533549 | STAB1 |
| 3 | 53170262 | 53201771 | PRKCD |
| 3 | 53855616 | 53874866 | IL17RB |
| 3 | 58293656 | 58385918 | PXK |
| 3 | 66511911 | 66633535 | LRIG1 |
| 3 | 69871322 | 70100176 | MITF |
| 3 | 71087426 | 71715830 | FOXP1 |
| 3 | 71903496 | 71916902 | PROK2 |
| 3 | 87391472 | 87408427 | POU1F1 |
| 3 | 89239363 | 89613972 | EPHA3 |
| 3 | 95074646 | 95175395 | PROS1 |
| 3 | 103029546 | 103062555 | NFKBIZ |
| 3 | 106568402 | 106778433 | ALCAM |
| 3 | 106859799 | 107070577 | CBLB |
| 3 | 109244630 | 109292625 | CD47 |
| 3 | 112743615 | 112853895 | CD96 |
| 3 | 113534605 | 113564346 | CD200 |
| 3 | 113665502 | 113701098 | BTLA |
| 3 | 114124221 | 114176627 | CD200R1 |
| 3 | 115330246 | 115380589 | DRD3 |
| 3 | 116825141 | 116922842 | GAP43 |
| 3 | 120725831 | 120761171 | CD80 |
| 3 | 120982020 | 121020021 | NR1I2 |
| 3 | 121028237 | 121295203 | GSK3B |
| 3 | 123256910 | 123322672 | CD86 |
| 3 | 123385219 | 123488032 | CASR |
| 3 | 124813834 | 125085839 | MYLK |
| 3 | 125964485 | 126088834 | ITGB5 |
| 3 | 128190191 | 128238918 | PLXNA1 |
| 3 | 128890600 | 129024741 | MGLL |
| 3 | 129680961 | 129694718 | GATA2 |
| 3 | 133518948 | 133560290 | ACPP |
| 3 | 133798783 | 133804070 | CCRL1 |
| 3 | 134947924 | 134980325 | TF |
| 3 | 138063762 | 138150657 | NCK1 |
| 3 | 139856922 | 139960875 | PIK3CB |
| 3 | 144321357 | 144324500 | CHST2 |
| 3 | 147715657 | 147745186 | PLSCR1 |
| 3 | 149898347 | 149943480 | AGTR1 |
| 3 | 152412594 | 152478920 | P2RY14 |
| 3 | 156280129 | 156384212 | MME |
| 3 | 158513627 | 158630410 | VIPR2 |
| 3 | 158637300 | 158644069 | PTX3 |
| 3 | 159771677 | 159805875 | MLF1 |
| 3 | 161189322 | 161196499 | IL12A |
| 3 | 170285243 | 170346787 | EVI1 |
| 3 | 170349579 | 170864168 | MDS1 |
| 3 | 171422913 | 171506464 | PRKCI |
| 3 | 171558209 | 171593226 | SKIL |
| 3 | 172801344 | 172938566 | PLD1 |
| 3 | 173645645 | 173648897 | GHSR |
| 3 | 173706158 | 173723963 | TNFSF10 |
| 3 | 180349004 | 180435189 | PIK3CA |
| 3 | 185572466 | 185578626 | THPO |
| 3 | 185580554 | 185590311 | CHRD |
| 3 | 187813580 | 187821799 | AHSG |
| 3 | 187917813 | 187944435 | KNG1 |
| 3 | 188043156 | 188058944 | ADIPOQ |
| 3 | 188131209 | 188279033 | ST6GAL1 |
| 3 | 188869389 | 188870895 | SST |
| 3 | 188921858 | 188946169 | BCL6 |
| 3 | 189413414 | 190080135 | LPP |
| 3 | 190831909 | 191097756 | TP63 |
| 3 | 191714584 | 191851995 | IL1RAP |
| 3 | 197074632 | 197120277 | ACK |
| 3 | 197260746 | 197293338 | TFRC |
| 3 | 198255819 | 198509844 | DLG1 |
| 4 | 1150722 | 1156597 | SPON2 |
| 4 | 1195227 | 1232908 | CTBP1 |
| 4 | 1273671 | 1323924 | MAEA |
| 4 | 2790338 | 2810712 | SH3BP2 |
| 4 | 3738093 | 3740049 | ADRA2C |
| 4 | 4471598 | 4594676 | STX18 |
| 4 | 15313738 | 15342893 | BST1 |
| 4 | 15389028 | 15459804 | CD38 |
| 4 | 19864332 | 20229886 | SLIT2 |
| 4 | 25930429 | 26042376 | RBPJ |
| 4 | 38474271 | 38482807 | TLR1 |
| 4 | 38504802 | 38507555 | TLR6 |
| 4 | 39874996 | 39922248 | RHOH |
| 4 | 47763517 | 47831030 | TXK |
| 4 | 47832556 | 47966571 | TEC |
| 4 | 54790203 | 54859168 | PDGFRA |
| 4 | 55218917 | 55301612 | KIT |
| 4 | 55639406 | 55686519 | KDR |
| 4 | 56156161 | 56197222 | NMU |
| 4 | 68107040 | 68155206 | STAP1 |
| 4 | 68285700 | 68304399 | GNRHR |
| 4 | 72826276 | 72868752 | GC |
| 4 | 74488869 | 74505996 | ALB |
| 4 | 74825138 | 74828295 | IL8 |
| 4 | 74921276 | 74923340 | CXCL6 |
| 4 | 74953972 | 74955817 | CXCL1 |
| 4 | 75065659 | 75066541 | PF4 |
| 4 | 75071621 | 75072764 | PPBP |
| 4 | 75080223 | 75083280 | CXCL5 |
| 4 | 75121177 | 75123354 | CXCL3 |
| 4 | 75181619 | 75183776 | CXCL2 |
| 4 | 75449723 | 75473341 | EREG |
| 4 | 77141646 | 77147665 | CXCL9 |
| 4 | 77161296 | 77163674 | CXCL10 |
| 4 | 77173865 | 77176257 | CXCL11 |
| 4 | 78651929 | 78752010 | CXCL13 |
| 4 | 79691765 | 79750627 | ANXA3 |
| 4 | 87156656 | 87593307 | MAPK10 |
| 4 | 88147163 | 88281214 | AFF1 |
| 4 | 89115825 | 89123586 | SPP1 |
| 4 | 89230440 | 89299035 | ABCG2 |
| 4 | 95438729 | 95483050 | PGDS |
| 4 | 100020235 | 100070139 | EIF4E |
| 4 | 100715003 | 100763649 | MTTP |
| 4 | 100957012 | 101010334 | DAPP1 |
| 4 | 102163609 | 102487376 | PPP3CA |
| 4 | 103641517 | 103757506 | NFKB1 |
| 4 | 107457123 | 107489020 | AIMP1 |
| 4 | 109188149 | 109309027 | LEF1 |
| 4 | 110829233 | 110844078 | CASP6 |
| 4 | 111053498 | 111152868 | EGF |
| 4 | 114190318 | 114524334 | ANK2 |
| 4 | 122808597 | 122837626 | ANXA5 |
| 4 | 122957974 | 122964330 | CCNA2 |
| 4 | 123519617 | 123570389 | ADAD1 |
| 4 | 123592075 | 123597100 | IL2 |
| 4 | 123753233 | 123761662 | IL21 |
| 4 | 123967312 | 124038840 | FGF2 |
| 4 | 142777203 | 142874061 | IL15 |
| 4 | 145786622 | 145879331 | HHIP |
| 4 | 146238605 | 146270125 | ABCE1 |
| 4 | 148621579 | 148685555 | EDNRA |
| 4 | 153461859 | 153675622 | FBXW7 |
| 4 | 154824890 | 154846690 | TLR2 |
| 4 | 155703595 | 155711686 | FGB |
| 4 | 155723729 | 155731347 | FGA |
| 4 | 155744736 | 155753352 | FGG |
| 4 | 157902213 | 158111996 | PDGFC |
| 4 | 158513627 | 158630410 | VIPR2 |
| 4 | 164464566 | 164473198 | NPY1R |
| 4 | 177841684 | 177950889 | VEGFC |
| 4 | 185545909 | 185632697 | IRF2 |
| 4 | 185785845 | 185807623 | CASP3 |
| 4 | 187227302 | 187243244 | TLR3 |
| 4 | 187385665 | 187416618 | KLKB1 |
| 5 | 1306286 | 1348162 | TERT |
| 5 | 35892747 | 35912678 | IL7R |
| 5 | 36642447 | 36724191 | SLC1A3 |
| 5 | 37851509 | 37875539 | GDNF |
| 5 | 38510822 | 38631253 | LIFR |
| 5 | 38881892 | 38970159 | OSMR |
| 5 | 39141114 | 39255424 | FYB |
| 5 | 39320764 | 39400412 | C9 |
| 5 | 40715788 | 40729592 | PTGER4 |
| 5 | 40945355 | 41018796 | C7 |
| 5 | 41178092 | 41249369 | C6 |
| 5 | 43417357 | 43448245 | CCL28 |
| 5 | 52119892 | 52285241 | ITGA1 |
| 5 | 52320912 | 52426365 | ITGA2 |
| 5 | 54434229 | 54441815 | GZMA |
| 5 | 55272451 | 55326520 | IL6ST |
| 5 | 58302467 | 58918032 | PDE4D |
| 5 | 63292034 | 63293302 | HTR1A |
| 5 | 66513871 | 66528368 | CD180 |
| 5 | 67558217 | 67633403 | PIK3R1 |
| 5 | 68498668 | 68509822 | CCNB1 |
| 5 | 68823874 | 68885886 | OCLN |
| 5 | 70300065 | 70356697 | NAIP |
| 5 | 74016766 | 74052867 | HEXB |
| 5 | 74668854 | 74693680 | HMGCR |
| 5 | 75947063 | 75954996 | F2RL2 |
| 5 | 76047546 | 76067054 | F2R |
| 5 | 76150609 | 76166895 | F2RL1 |
| 5 | 77333906 | 77626284 | AP3B1 |
| 5 | 80292313 | 80557709 | RASGRF2 |
| 5 | 82409072 | 82685333 | XRCC4 |
| 5 | 86599906 | 86723488 | RASA1 |
| 5 | 96023696 | 96136139 | CAST |
| 5 | 96122270 | 96169648 | ERAP1 |
| 5 | 96241023 | 96279367 | ERAP2 |
| 5 | 108111421 | 108551272 | FER |
| 5 | 110435288 | 110441622 | TSLP |
| 5 | 112101482 | 112209834 | APC |
| 5 | 114942246 | 114989610 | TICAM2 |
| 5 | 115168330 | 115180304 | CDO1 |
| 5 | 131424245 | 131426795 | IL3 |
| 5 | 131437383 | 131439757 | CSF2 |
| 5 | 131846683 | 131854326 | IRF1 |
| 5 | 131905034 | 131907113 | IL5 |
| 5 | 132021763 | 132024700 | IL13 |
| 5 | 132037271 | 132046267 | IL4 |
| 5 | 132415560 | 132468607 | HSPA4 |
| 5 | 133478300 | 133511818 | TCF7 |
| 5 | 133520467 | 133540583 | SKP1 |
| 5 | 134102104 | 134115740 | CAMLG |
| 5 | 134934274 | 134942868 | CXCL14 |
| 5 | 135255833 | 135259415 | IL9 |
| 5 | 137829079 | 137832903 | EGR1 |
| 5 | 139473891 | 139476504 | PURA |
| 5 | 139991500 | 139993439 | CD14 |
| 5 | 140007567 | 140022249 | IK |
| 5 | 140033674 | 140051155 | HARS |
| 5 | 140874772 | 140978747 | DIAPH1 |
| 5 | 141468507 | 141513128 | NDFIP1 |
| 5 | 141953306 | 142045812 | FGF1 |
| 5 | 142637688 | 142795270 | NR3C1 |
| 5 | 147423758 | 147497120 | SPINK5 |
| 5 | 148186348 | 148188381 | ADRB2 |
| 5 | 148734024 | 148739031 | IL17B |
| 5 | 148855037 | 148911200 | CSNK1A1 |
| 5 | 149413050 | 149473128 | CSF1R |
| 5 | 149473594 | 149515615 | PDGFRB |
| 5 | 149761393 | 149772525 | CD74 |
| 5 | 149880622 | 149917965 | NDST1 |
| 5 | 150389700 | 150441190 | TNIP1 |
| 5 | 156278948 | 156322844 | TIMD4 |
| 5 | 156389014 | 156418548 | HAVCR1 |
| 5 | 156445420 | 156468716 | HAVCR2 |
| 5 | 156498016 | 156502453 | MED7 |
| 5 | 156540484 | 156614687 | ITK |
| 5 | 158058005 | 158459347 | EBF1 |
| 5 | 158513627 | 158630410 | VIPR2 |
| 5 | 158674368 | 158690059 | IL12B |
| 5 | 159276317 | 159332597 | ADRA1B |
| 5 | 159781442 | 159788323 | PTTG1 |
| 5 | 168996870 | 169442959 | DOCK2 |
| 5 | 169607666 | 169657400 | LCP2 |
| 5 | 170747402 | 170770492 | NPM1 |
| 5 | 172127706 | 172130809 | DUSP1 |
| 5 | 175042317 | 175044162 | HRH2 |
| 5 | 176663440 | 176666555 | PRELID1 |
| 5 | 176666818 | 176671487 | MXD3 |
| 5 | 176761744 | 176769183 | F12 |
| 5 | 176786293 | 176802436 | GRK6 |
| 5 | 176863357 | 176869459 | DOK3 |
| 5 | 179153591 | 179156118 | LTC4S |
| 5 | 179595389 | 179640216 | MAPK9 |
| 6 | 336759 | 356193 | IRF4 |
| 6 | 2945229 | 2964993 | NQO2 |
| 6 | 3022056 | 3060418 | RIPK1 |
| 6 | 6089309 | 6265923 | F13A1 |
| 6 | 6533932 | 6600215 | LY86 |
| 6 | 7672009 | 7826960 | BMP6 |
| 6 | 11291519 | 11340884 | NEDD9 |
| 6 | 12398644 | 12404761 | EDN1 |
| 6 | 14225843 | 14245125 | CD83 |
| 6 | 15354505 | 15630231 | JARID2 |
| 6 | 21701950 | 21706826 | SOX4 |
| 6 | 22395458 | 22405709 | PRL |
| 6 | 26163946 | 26164678 | HIST1H1C |
| 6 | 26609427 | 26618629 | BTN1A1 |
| 6 | 29663661 | 29664724 | OR2H2 |
| 6 | 29732787 | 29748126 | MOG |
| 6 | 29903496 | 29906856 | HLA-G |
| 6 | 30018309 | 30021632 | HLA-A |
| 6 | 30335352 | 31347834 | HLA-C |
| 6 | 30565249 | 30569064 | HLA-E |
| 6 | 30647148 | 30667286 | ABCF1 |
| 6 | 30818954 | 30820306 | IER3 |
| 6 | 30959839 | 30975910 | DDR1 |
| 6 | 31429629 | 31432914 | HLA-B |
| 6 | 31538937 | 31541460 | HCP5 |
| 6 | 31648071 | 31650077 | LTA |
| 6 | 31651328 | 31654089 | TNF |
| 6 | 31656316 | 31658181 | LTB |
| 6 | 31661949 | 31664664 | LST1 |
| 6 | 31664650 | 31668741 | NCR3 |
| 6 | 31691011 | 31692775 | AIF1 |
| 6 | 31891315 | 31893696 | HSPA1A |
| 6 | 32003472 | 32021427 | C2 |
| 6 | 32021751 | 32027839 | CFB |
| 6 | 32057812 | 32078435 | C4B |
| 6 | 32090549 | 32111173 | C4A |
| 6 | 32229278 | 32239429 | PPT2 |
| 6 | 32256723 | 32260001 | AGER |
| 6 | 32266521 | 32271278 | GPSM3 |
| 6 | 32270598 | 32299822 | NOTCH4 |
| 6 | 32470491 | 32482878 | BTNL2 |
| 6 | 32515624 | 32520799 | HLA-DRA |
| 6 | 32654526 | 32665559 | HLA-DRB1 |
| 6 | 32713160 | 32823197 | HLA-DQA1 |
| 6 | 32897587 | 32914525 | TAP2 |
| 6 | 32916476 | 32920690 | PSMB8 |
| 6 | 32920964 | 32929726 | TAP1 |
| 6 | 32929915 | 32935604 | PSMB9 |
| 6 | 33010393 | 33016795 | HLA-DMB |
| 6 | 33024373 | 33028831 | HLA-DMA |
| 6 | 33079939 | 33085367 | HLA-DOA |
| 6 | 33365355 | 33366689 | PFDN6 |
| 6 | 33367415 | 33374716 | RGL2 |
| 6 | 33375451 | 33389967 | TAPBP |
| 6 | 33648301 | 33656048 | BAK1 |
| 6 | 33697321 | 33772316 | ITPR3 |
| 6 | 34312627 | 34321985 | HMGA1 |
| 6 | 35373572 | 35397526 | DEF6 |
| 6 | 35418312 | 35503931 | PPARD |
| 6 | 36103550 | 36186513 | MAPK14 |
| 6 | 36754464 | 36763086 | CDKN1A |
| 6 | 37245963 | 37251180 | PIM1 |
| 6 | 41148687 | 41174713 | NFYA |
| 6 | 41234230 | 41238892 | TREM2 |
| 6 | 41351689 | 41362435 | TREM1 |
| 6 | 41411504 | 41426593 | NCR2 |
| 6 | 42010649 | 42017530 | CCND3 |
| 6 | 42991704 | 43001551 | PTCRA |
| 6 | 43246897 | 43257221 | SRF |
| 6 | 43845930 | 43862199 | VEGFA |
| 6 | 44295219 | 44309866 | SLC29A1 |
| 6 | 44322826 | 44329592 | HSP90AB1 |
| 6 | 45404031 | 45626796 | RUNX2 |
| 6 | 46780237 | 46811055 | PLA2G7 |
| 6 | 46869085 | 46915473 | MEP1A |
| 6 | 47307227 | 47385639 | TNFRSF21 |
| 6 | 52159143 | 52163395 | IL17A |
| 6 | 86216527 | 86262215 | NT5E |
| 6 | 88906305 | 88911775 | CNR1 |
| 6 | 91282073 | 91353628 | MAP3K7 |
| 6 | 105832200 | 105957662 | PREP |
| 6 | 106640887 | 106664502 | PRDM1 |
| 6 | 108987718 | 109108646 | FOXO3 |
| 6 | 109794415 | 109810340 | CD164 |
| 6 | 110527714 | 110607900 | WASF1 |
| 6 | 111726926 | 111911107 | REV3L |
| 6 | 111986837 | 112034014 | TRAF3IP2 |
| 6 | 112089179 | 112301320 | FYN |
| 6 | 114364021 | 114399047 | HDAC2 |
| 6 | 121798443 | 121812572 | GJA1 |
| 6 | 129245978 | 129879401 | LAMA2 |
| 6 | 130381426 | 130504277 | L3MBTL3 |
| 6 | 132000134 | 132110242 | ENPP3 |
| 6 | 132311011 | 132314211 | CTGF |
| 6 | 133044424 | 133076881 | VNN1 |
| 6 | 135544145 | 135582002 | MYB |
| 6 | 136919880 | 137155349 | MAP3K5 |
| 6 | 137506650 | 137536478 | IL22RA2 |
| 6 | 137560314 | 137582200 | IFNGR1 |
| 6 | 138230273 | 138246138 | TNFAIP3 |
| 6 | 138453618 | 138470280 | PERP |
| 6 | 143114297 | 143308031 | HIVEP2 |
| 6 | 150304828 | 150312059 | ULBP2 |
| 6 | 150427436 | 150431895 | ULBP3 |
| 6 | 152170378 | 152466099 | ESR1 |
| 6 | 153113625 | 153122591 | VIP |
| 6 | 154402135 | 154609693 | OPRM1 |
| 6 | 158513627 | 158630410 | VIPR2 |
| 6 | 160020140 | 160034343 | SOD2 |
| 6 | 160310120 | 160447573 | IGF2R |
| 6 | 160872505 | 161007397 | LPA |
| 6 | 161043272 | 161094328 | PLG |
| 6 | 167456246 | 167472617 | CCR6 |
| 6 | 169357800 | 169396062 | THBS2 |
| 6 | 170433220 | 170441622 | DLL1 |
| 7 | 555912 | 718659 | PRKAR1B |
| 7 | 1821953 | 2239109 | MAD1L1 |
| 7 | 2526004 | 2535334 | LFNG |
| 7 | 2734268 | 2850485 | GNA12 |
| 7 | 2912308 | 3049996 | CARD11 |
| 7 | 6029990 | 6065302 | EIF2AK1 |
| 7 | 6380650 | 6410122 | RAC1 |
| 7 | 17304831 | 17352297 | AHR |
| 7 | 18501893 | 19003509 | HDAC7 |
| 7 | 18501893 | 19003509 | HDAC9 |
| 7 | 22733344 | 22738141 | IL6 |
| 7 | 24290333 | 24298002 | NPY |
| 7 | 25124801 | 25131480 | CYCS |
| 7 | 27112334 | 27133164 | HOXA3 |
| 7 | 27168582 | 27171674 | HOXA9 |
| 7 | 27176735 | 27186368 | HOXA10 |
| 7 | 30430674 | 30484790 | NOD1 |
| 7 | 30658724 | 30688421 | CRHR2 |
| 7 | 36519134 | 36730550 | AOAH |
| 7 | 36860485 | 37455036 | ELMO1 |
| 7 | 38265768 | 38279773 | TCRg |
| 7 | 38389831 | 38637545 | AMPH |
| 7 | 41695127 | 41709231 | INHBA |
| 7 | 44078373 | 44088607 | POLM |
| 7 | 44802765 | 44809240 | PPIA |
| 7 | 50314923 | 50438053 | IKZF1 |
| 7 | 55054218 | 55242524 | EGFR |
| 7 | 65217239 | 65256986 | CRCP |
| 7 | 65307748 | 65462864 | TPST1 |
| 7 | 65843154 | 65913881 | RABGEF1 |
| 7 | 66090124 | 66098023 | SBDS |
| 7 | 73080362 | 73122172 | ELN |
| 7 | 73262022 | 73282099 | LAT2 |
| 7 | 73826244 | 73841594 | NCF1 |
| 7 | 75236777 | 75257000 | CCL26 |
| 7 | 75279051 | 75280969 | CCL24 |
| 7 | 75864803 | 75909323 | ZP3 |
| 7 | 76660625 | 76667086 | FGL2 |
| 7 | 77004770 | 77107322 | PTPN12 |
| 7 | 80069458 | 80144259 | CD36 |
| 7 | 80209790 | 80386603 | SEMA3C |
| 7 | 81169380 | 81237388 | HGF |
| 7 | 83425600 | 83662153 | SEMA3A |
| 7 | 86619859 | 86663580 | DMTF1 |
| 7 | 86869296 | 86942955 | ABCB4 |
| 7 | 89678935 | 89704927 | STEAP2 |
| 7 | 94872110 | 94902320 | PON2 |
| 7 | 97199310 | 97207718 | TAC1 |
| 7 | 98844547 | 98855174 | S1PR1 |
| 7 | 98844547 | 98855174 | S1PR2 |
| 7 | 98844547 | 98855174 | S1PR3 |
| 7 | 98844547 | 98855174 | S1PR4 |
| 7 | 98844547 | 98855174 | S1PR5 |
| 7 | 100156358 | 100159257 | EPO |
| 7 | 100325551 | 100331477 | ACHE |
| 7 | 100557171 | 100569026 | SERPINE1 |
| 7 | 101715165 | 101748897 | SH2B2 |
| 7 | 105675967 | 105712874 | NAMPT |
| 7 | 106293159 | 106334821 | PIK3CG |
| 7 | 106596695 | 106630209 | HBP1 |
| 7 | 115952074 | 115988466 | CAV1 |
| 7 | 116907252 | 117095952 | CFTR |
| 7 | 123109236 | 123176352 | WASL |
| 7 | 123272458 | 123304765 | HYAL4 |
| 7 | 127668566 | 127684917 | LEP |
| 7 | 127819567 | 127837542 | IMPDH1 |
| 7 | 128365229 | 128377322 | IRF5 |
| 7 | 130835560 | 130891916 | PODXL |
| 7 | 136203938 | 136352311 | CHRM2 |
| 7 | 138378807 | 138445005 | ZC3HAV1 |
| 7 | 140080753 | 140271033 | BRAF |
| 7 | 141273625 | 141293252 | CLEC5A |
| 7 | 142262913 | 142278967 | EPHB6 |
| 7 | 142695523 | 142714906 | CASP2 |
| 7 | 142798327 | 142816107 | EPHA1 |
| 7 | 148135407 | 148212347 | EZH2 |
| 7 | 149666351 | 149669639 | RARRES2 |
| 7 | 150065383 | 150071669 | GIMAP5 |
| 7 | 150319079 | 150342608 | NOS3 |
| 7 | 150381831 | 150385929 | CDK5 |
| 7 | 155288318 | 155297728 | SHH |
| 7 | 158513627 | 158630410 | VIPR2 |
| 8 | 2782789 | 4839736 | CSMD1 |
| 8 | 6347600 | 6408172 | ANGPT2 |
| 8 | 6715510 | 6722939 | DEFB1 |
| 8 | 6769630 | 6771008 | SWAP70 |
| 8 | 6780754 | 6783196 | DEFA4 |
| 8 | 6822580 | 6863233 | DEFA1 |
| 8 | 6860804 | 6863226 | DEFA3 |
| 8 | 6900238 | 6901669 | DEFA5 |
| 8 | 7273825 | 7777590 | DEFB103A |
| 8 | 7273825 | 7777590 | DEFB3 |
| 8 | 7273900 | 7777515 | DEFB103B |
| 8 | 7315240 | 7736173 | DEFB104A |
| 8 | 7315240 | 7736173 | DEFB3 |
| 8 | 7332652 | 7718770 | DEFB105A |
| 8 | 7789608 | 7791646 | DEFB4 |
| 8 | 11388929 | 11459516 | BLK |
| 8 | 11599161 | 11654918 | GATA4 |
| 8 | 11737444 | 11763055 | CTSB |
| 8 | 16009758 | 16094671 | MSR1 |
| 8 | 17440664 | 17472352 | SLC7A2 |
| 8 | 21822330 | 21827151 | DOK2 |
| 8 | 22075112 | 22077928 | SFTPC |
| 8 | 22601118 | 22606760 | EGR3 |
| 8 | 22933592 | 22982637 | TNFRSF10B |
| 8 | 23016378 | 23030893 | TNFRSF10C |
| 8 | 23104915 | 23138584 | TNFRSF10A |
| 8 | 25332690 | 25338473 | GNRH1 |
| 8 | 26491337 | 26571610 | DPYSL2 |
| 8 | 26661583 | 26778839 | ADRA1A |
| 8 | 27224915 | 27372820 | PTK2B |
| 8 | 27404561 | 27458401 | EPHX2 |
| 8 | 27510368 | 27528244 | CLU |
| 8 | 28230567 | 28256785 | PNOC |
| 8 | 29249538 | 29264104 | DUSP4 |
| 8 | 37939672 | 37943341 | ADRB3 |
| 8 | 38973661 | 39081934 | ADAM9 |
| 8 | 41907426 | 42028635 | MYST3 |
| 8 | 42151392 | 42184351 | PLAT |
| 8 | 42247985 | 42309122 | IKBKB |
| 8 | 48812028 | 48813279 | CEBPD |
| 8 | 48848221 | 49035296 | PRKDC |
| 8 | 49992795 | 49996541 | SNAI2 |
| 8 | 54300828 | 54326747 | OPRK1 |
| 8 | 56954925 | 57085685 | LYN |
| 8 | 57516070 | 57521143 | PENK |
| 8 | 59880530 | 60194321 | TOX |
| 8 | 61753892 | 61942017 | CHD7 |
| 8 | 67251172 | 67253252 | CRH |
| 8 | 67636967 | 67687729 | MYBL1 |
| 8 | 75066143 | 75103859 | LY96 |
| 8 | 79807560 | 79880313 | IL7 |
| 8 | 82042602 | 82186858 | PAG1 |
| 8 | 82515120 | 82522274 | PMP2 |
| 8 | 82553489 | 82558004 | FABP4 |
| 8 | 87424109 | 87549292 | WWP1 |
| 8 | 90839109 | 90872432 | RIPK2 |
| 8 | 91014739 | 91066075 | NBN |
| 8 | 91140013 | 91164283 | CALB1 |
| 8 | 93040327 | 93176619 | RUNX1T1 |
| 8 | 95330662 | 95343733 | GEM |
| 8 | 96007376 | 96030767 | TP53INP1 |
| 8 | 101784319 | 101803491 | PABPC1 |
| 8 | 105421229 | 105438092 | TM7SF4 |
| 8 | 108330887 | 108579430 | ANGPT1 |
| 8 | 120004977 | 120033492 | TNFRSF11B |
| 8 | 120638499 | 120720287 | ENPP2 |
| 8 | 128817497 | 128822853 | MYC |
| 8 | 133948386 | 134216323 | TG |
| 8 | 134118155 | 134184479 | SLA |
| 8 | 134318595 | 134378680 | NDRG1 |
| 8 | 134540326 | 134653344 | ST3GAL1 |
| 8 | 141737682 | 142080514 | PTK2 |
| 8 | 143950776 | 143958238 | CYP11B1 |
| 8 | 144728098 | 144731636 | NAPRT1 |
| 8 | 145061308 | 145121531 | PLEC1 |
| 8 | 145486077 | 145509193 | HSF1 |
| 9 | 4975244 | 5117994 | JAK2 |
| 9 | 5289867 | 5294580 | RLN2 |
| 9 | 5440558 | 5458475 | CD274 |
| 9 | 5500569 | 5560398 | PDCD1LG2 |
| 9 | 5880908 | 5899822 | MLANA |
| 9 | 6231677 | 6247982 | IL33 |
| 9 | 12683448 | 12700249 | TYRP1 |
| 9 | 19366253 | 19370235 | RPS6 |
| 9 | 20334967 | 20612450 | MLLT3 |
| 9 | 21067104 | 21067943 | IFNB1 |
| 9 | 21130630 | 21132144 | IFNW1 |
| 9 | 21155635 | 21156619 | IFNA21 |
| 9 | 21176692 | 21177670 | IFNA4 |
| 9 | 21191233 | 21229990 | IFNA14 |
| 9 | 21191467 | 21192204 | IFNA7 |
| 9 | 21196179 | 21197142 | IFNA10 |
| 9 | 21206371 | 21207310 | IFNA16 |
| 9 | 21217241 | 21218221 | IFNA |
| 9 | 21217241 | 21218221 | IFNA17 |
| 9 | 21294686 | 21295255 | IFNA5 |
| 9 | 21340318 | 21340886 | IFNA6 |
| 9 | 21374254 | 21375396 | IFNA |
| 9 | 21374254 | 21375396 | IFNA2 |
| 9 | 21399145 | 21400184 | IFNA8 |
| 9 | 21430439 | 21431315 | IFNA1 |
| 9 | 21957751 | 21984490 | CDKN2A |
| 9 | 21992905 | 21999312 | CDKN2B |
| 9 | 26894517 | 26937139 | PLAA |
| 9 | 27514311 | 27516494 | IFNK |
| 9 | 27938527 | 28709303 | LINGO2 |
| 9 | 32445300 | 32516322 | DDX58 |
| 9 | 33100641 | 33157231 | B4GALT1 |
| 9 | 33280509 | 33361154 | NFX1 |
| 9 | 33785558 | 33789228 | PRSS3 |
| 9 | 34643931 | 34651883 | IL11RA |
| 9 | 34651892 | 34652689 | CCL27 |
| 9 | 34679568 | 34681274 | CCL19 |
| 9 | 34699003 | 34700147 | CCL21 |
| 9 | 35599975 | 35608408 | CD72 |
| 9 | 35639296 | 35640947 | SIT1 |
| 9 | 36828530 | 37024476 | PAX5 |
| 9 | 37905895 | 38059210 | SHB |
| 9 | 72189334 | 72219393 | KLF9 |
| 9 | 74956600 | 74975127 | ANXA1 |
| 9 | 77695440 | 77998155 | PCSK5 |
| 9 | 78263965 | 78312150 | GCNT1 |
| 9 | 79525019 | 79836012 | GNAQ |
| 9 | 89530793 | 89536204 | CTSL1 |
| 9 | 90795662 | 90809744 | S1PR3 |
| 9 | 91181971 | 91284431 | SEMA4D |
| 9 | 91409747 | 91411287 | GADD45G |
| 9 | 92603890 | 92698298 | SYK |
| 9 | 93211149 | 93225965 | NFIL3 |
| 9 | 96901158 | 97119812 | FANCC |
| 9 | 98834760 | 98841360 | CTSL2 |
| 9 | 99477012 | 99499460 | XPA |
| 9 | 100907232 | 100956292 | TGFBR1 |
| 9 | 103393719 | 103397104 | PPP3R2 |
| 9 | 106583106 | 106730257 | ABCA1 |
| 9 | 109286955 | 109291576 | KLF4 |
| 9 | 110669621 | 110736217 | IKBKAP |
| 9 | 112046130 | 112058599 | TXN |
| 9 | 115246831 | 115399838 | RGS3 |
| 9 | 115862230 | 115880536 | AMBP |
| 9 | 116125156 | 116128578 | ORM1 |
| 9 | 116131889 | 116135355 | ORM2 |
| 9 | 116591433 | 116608229 | TNFSF15 |
| 9 | 116704944 | 116732591 | TNFSF8 |
| 9 | 116822633 | 116920260 | TNC |
| 9 | 119506430 | 119519585 | TLR4 |
| 9 | 122704492 | 122728994 | TRAF1 |
| 9 | 122754436 | 122852375 | C5 |
| 9 | 123070200 | 123134941 | GSN |
| 9 | 124173049 | 124197802 | PTGS1 |
| 9 | 126283336 | 126309520 | NR5A1 |
| 9 | 127036954 | 127043430 | HSPA5 |
| 9 | 129951552 | 129955556 | LCN2 |
| 9 | 130222937 | 130239444 | CERCAM |
| 9 | 131540432 | 131555165 | PTGES |
| 9 | 132309914 | 132366481 | ASS1 |
| 9 | 132579088 | 132752881 | ABL1 |
| 9 | 133441978 | 133602746 | RAPGEF1 |
| 9 | 134756557 | 134809841 | TSC1 |
| 9 | 134843918 | 134856903 | GFI1B |
| 9 | 134962928 | 135014409 | RALGDS |
| 9 | 135491305 | 135514287 | DBH |
| 9 | 135616837 | 135847226 | VAV2 |
| 9 | 136358230 | 136472253 | RXRA |
| 9 | 137593424 | 137598444 | PAEP |
| 9 | 138378229 | 138387929 | CARD9 |
| 10 | 6034340 | 6060148 | IL15RA |
| 10 | 6093511 | 6144278 | IL2RA |
| 10 | 6284900 | 6317498 | PFKFB3 |
| 10 | 6509110 | 6662244 | PRKCQ |
| 10 | 8136672 | 8157168 | GATA3 |
| 10 | 14988878 | 15036100 | DCLRE1C |
| 10 | 17726129 | 17797913 | STAM |
| 10 | 17891367 | 18240097 | MRC1 |
| 10 | 21863107 | 22072561 | MLLT10 |
| 10 | 22085483 | 22332656 | DNAJC1 |
| 10 | 22650145 | 22660190 | BMI1 |
| 10 | 26545599 | 26633492 | GAD2 |
| 10 | 31648147 | 31856740 | ZEB1 |
| 10 | 33229326 | 33287204 | ITGB1 |
| 10 | 33506431 | 33663839 | NRP1 |
| 10 | 44185612 | 44200548 | CXCL12 |
| 10 | 45189634 | 45261567 | ALOX5 |
| 10 | 46503539 | 46508325 | PP1 |
| 10 | 48001494 | 48010997 | RBP3 |
| 10 | 49279692 | 49313188 | MAPK8 |
| 10 | 50696332 | 51440263 | PARG |
| 10 | 51735351 | 52053743 | SGMS1 |
| 10 | 52504298 | 53725280 | PRKG1 |
| 10 | 53744046 | 53747422 | DKK1 |
| 10 | 54195146 | 54201466 | MBL2 |
| 10 | 62208241 | 62223930 | CDC2 |
| 10 | 64241762 | 64246133 | EGR2 |
| 10 | 70517833 | 70534571 | SRGN |
| 10 | 71632591 | 71663196 | PP1 |
| 10 | 72027109 | 72032537 | PRF1 |
| 10 | 73394125 | 73443318 | CHST3 |
| 10 | 73797103 | 74055905 | CBARA1 |
| 10 | 74866617 | 74925760 | PPP3CB |
| 10 | 75231674 | 75241348 | NDST2 |
| 10 | 75340895 | 75347260 | PLAU |
| 10 | 81040717 | 81363920 | SFTPA1 |
| 10 | 81687477 | 81698841 | SFTPD |
| 10 | 81904859 | 81955308 | ANXA11 |
| 10 | 82021556 | 82039414 | MAT1A |
| 10 | 88506375 | 88674924 | BMPR1A |
| 10 | 89613174 | 89718511 | PTEN |
| 10 | 90740267 | 90765521 | FAS |
| 10 | 90963311 | 91001640 | LIPA |
| 10 | 92661836 | 92671012 | ANKRD1 |
| 10 | 96295563 | 96351845 | HELLS |
| 10 | 97505905 | 97616454 | ENTPD1 |
| 10 | 97941452 | 98021316 | BLNK |
| 10 | 98343060 | 98470269 | PIK3AP1 |
| 10 | 99176016 | 99183187 | PGAM1 |
| 10 | 100165945 | 100196694 | HPS1 |
| 10 | 101282699 | 101286268 | NKX2-3 |
| 10 | 101792054 | 101831632 | CPN1 |
| 10 | 101938114 | 101979334 | CHUK |
| 10 | 102880251 | 102887526 | TLX1 |
| 10 | 104144218 | 104152270 | NFKB2 |
| 10 | 105781035 | 105835628 | COL17A1 |
| 10 | 112826910 | 112830560 | ADRA2A |
| 10 | 115428924 | 115480652 | CASP7 |
| 10 | 115793795 | 115796655 | ADRB1 |
| 10 | 116044574 | 116154505 | AFAP1L2 |
| 10 | 117812942 | 118022966 | GFRA1 |
| 10 | 120917205 | 120928335 | PRDX3 |
| 10 | 121322969 | 121346531 | TIAL1 |
| 10 | 124310170 | 124393242 | DMBT1 |
| 10 | 127693414 | 128067055 | ADAM12 |
| 10 | 128584012 | 129140769 | DOCK1 |
| 10 | 135010900 | 135016177 | PRAP1 |
| 11 | 297914 | 299392 | IFITM2 |
| 11 | 309668 | 311050 | IFITM3 |
| 11 | 395715 | 407397 | SIGIRR |
| 11 | 522242 | 525550 | HRAS |
| 11 | 602555 | 605999 | IRF7 |
| 11 | 616312 | 617173 | SCT |
| 11 | 822951 | 828834 | CD151 |
| 11 | 1730560 | 1741798 | CTSD |
| 11 | 1830775 | 1870068 | LSP1 |
| 11 | 2106923 | 2139015 | IGF2 |
| 11 | 2124432 | 2139015 | INS |
| 11 | 2279818 | 2296006 | TSPAN32 |
| 11 | 2355122 | 2375225 | CD81 |
| 11 | 3652816 | 3775468 | NUP98 |
| 11 | 3833508 | 4071015 | STIM1 |
| 11 | 4362702 | 4371502 | TRIM21 |
| 11 | 5203271 | 5204877 | HBB |
| 11 | 5667494 | 5688668 | TRIM22 |
| 11 | 6368230 | 6372801 | SMPD1 |
| 11 | 6409017 | 6418769 | HPX |
| 11 | 8202432 | 8241982 | LMO1 |
| 11 | 9642203 | 9731081 | SWAP70 |
| 11 | 10283217 | 10285499 | ADM |
| 11 | 11941120 | 11987493 | DKK3 |
| 11 | 13255900 | 13365386 | ARNTL |
| 11 | 14944791 | 14950408 | CALCA |
| 11 | 15051721 | 15056753 | CALCB |
| 11 | 17697685 | 17700253 | MYOD1 |
| 11 | 18244347 | 18248102 | SAA1 |
| 11 | 31767034 | 31789455 | PAX6 |
| 11 | 32365900 | 32413663 | WT1 |
| 11 | 33681133 | 33714600 | CD59 |
| 11 | 34417053 | 34450179 | CAT |
| 11 | 35116992 | 35210522 | CD44 |
| 11 | 35229328 | 35397681 | SLC1A2 |
| 11 | 36467298 | 36488398 | TRAF6 |
| 11 | 36546138 | 36557871 | RAG1 |
| 11 | 36570070 | 36576362 | RAG2 |
| 11 | 44543716 | 44597889 | CD82 |
| 11 | 45627002 | 45643748 | CHST1 |
| 11 | 45783911 | 45791142 | SLC35C1 |
| 11 | 46311314 | 46358680 | DGKZ |
| 11 | 46359193 | 46361950 | MDK |
| 11 | 46363215 | 46364683 | CHRM4 |
| 11 | 46655207 | 46678696 | ARHGAP1 |
| 11 | 46697330 | 46717631 | F2 |
| 11 | 47332984 | 47356703 | SPI1 |
| 11 | 47958688 | 48146246 | PTPRJ |
| 11 | 56900818 | 56905199 | PRG3 |
| 11 | 56911410 | 56914706 | PRG2 |
| 11 | 57121602 | 57138902 | SERPING1 |
| 11 | 58146720 | 58149776 | CNTF |
| 11 | 59612712 | 59622590 | MS4A2 |
| 11 | 59979857 | 59994800 | MS4A1 |
| 11 | 60374982 | 60380020 | GPR44 |
| 11 | 60495749 | 60544422 | CD6 |
| 11 | 60626542 | 60651897 | CD5 |
| 11 | 61039363 | 61104874 | SYT7 |
| 11 | 61488334 | 61491708 | FTH1 |
| 11 | 61820329 | 61823112 | SCGB1D4 |
| 11 | 61943098 | 61947242 | SCGB1A1 |
| 11 | 62432727 | 62445588 | CHRM1 |
| 11 | 63060855 | 63070505 | RARRES3 |
| 11 | 63775697 | 63791603 | PLCB3 |
| 11 | 63793877 | 63808740 | BAD |
| 11 | 63842144 | 63845858 | PRDX5 |
| 11 | 64250959 | 64269504 | RASGRP2 |
| 11 | 64313185 | 64327289 | MAP4K2 |
| 11 | 64327571 | 64335342 | MEN1 |
| 11 | 65162170 | 65174965 | SIPA1 |
| 11 | 65178392 | 65186951 | RELA |
| 11 | 65403859 | 65407788 | CTSW |
| 11 | 65416267 | 65424573 | FOSL1 |
| 11 | 65485735 | 65504182 | SART1 |
| 11 | 65526125 | 65528192 | BANF1 |
| 11 | 65886568 | 65895867 | SLC29A2 |
| 11 | 66580896 | 66596059 | RHOD |
| 11 | 66790668 | 66810933 | ADRBK1 |
| 11 | 66888216 | 66897782 | CLCF1 |
| 11 | 66959556 | 66961729 | PTPRCAP |
| 11 | 67007096 | 67015150 | AIP |
| 11 | 67107861 | 67110699 | GSTP1 |
| 11 | 67515150 | 67528169 | UNC93B1 |
| 11 | 67563058 | 67574941 | TCIRG1 |
| 11 | 67576903 | 67645434 | CHKA |
| 11 | 67836683 | 67973319 | LRP5 |
| 11 | 69165053 | 69178422 | CCND1 |
| 11 | 69333917 | 69343129 | FGF3 |
| 11 | 69726916 | 69731134 | FADD |
| 11 | 70823104 | 70837125 | DHCR7 |
| 11 | 71387605 | 71391222 | IL18BP |
| 11 | 72765052 | 72786166 | RELT |
| 11 | 73363363 | 73371537 | UCP2 |
| 11 | 74654129 | 74740521 | ARRB1 |
| 11 | 76516963 | 76603932 | MYO7A |
| 11 | 76710708 | 76862581 | PAK1 |
| 11 | 77603990 | 77806414 | GAB2 |
| 11 | 85046517 | 85053821 | CREBZF |
| 11 | 85346133 | 85457756 | PICALM |
| 11 | 86189138 | 86199921 | PRSS23 |
| 11 | 87666408 | 87710586 | CTSC |
| 11 | 87880625 | 88420838 | GRM5 |
| 11 | 88550687 | 88668574 | TYR |
| 11 | 88699160 | 88864301 | NOX4 |
| 11 | 93501741 | 93554783 | PANX1 |
| 11 | 93916774 | 93922711 | FUT4 |
| 11 | 100414312 | 100506465 | PGR |
| 11 | 101693403 | 101713674 | BIRC3 |
| 11 | 101896449 | 101906688 | MMP7 |
| 11 | 102088541 | 102100868 | MMP8 |
| 11 | 102211737 | 102219552 | MMP3 |
| 11 | 102238673 | 102250922 | MMP12 |
| 11 | 102318934 | 102331672 | MMP13 |
| 11 | 104318804 | 104344535 | CASP4 |
| 11 | 104370180 | 104384909 | CASP5 |
| 11 | 104401451 | 104411067 | CASP1 |
| 11 | 107598768 | 107745036 | ATM |
| 11 | 110728189 | 110755627 | POU2AF1 |
| 11 | 111158129 | 111247515 | ALG9 |
| 11 | 111288708 | 111302805 | HSPB2 |
| 11 | 111519185 | 111540050 | IL18 |
| 11 | 112785527 | 112851091 | DRD2 |
| 11 | 113435640 | 113626604 | ZBTB16 |
| 11 | 114550226 | 114880325 | CADM1 |
| 11 | 116196627 | 116199221 | APOA4 |
| 11 | 116211678 | 116213548 | APOA1 |
| 11 | 117362318 | 117377403 | IL10RA |
| 11 | 117680661 | 117692099 | CD3E |
| 11 | 117714998 | 117718669 | CD3D |
| 11 | 117720310 | 117729261 | CD3G |
| 11 | 117812414 | 117901144 | MLL |
| 11 | 118259776 | 118272180 | CXCR5 |
| 11 | 118400275 | 118406800 | SLC37A4 |
| 11 | 118420109 | 118433122 | HYOU1 |
| 11 | 118582199 | 118684068 | CBL |
| 11 | 118794097 | 118799064 | THY1 |
| 11 | 122214464 | 122248555 | CRTAM |
| 11 | 124048949 | 124069895 | SPA17 |
| 11 | 125001546 | 125030847 | CHEK1 |
| 11 | 125658191 | 125670038 | TIRAP |
| 11 | 127833871 | 127897371 | ETS1 |
| 11 | 128069198 | 128187520 | FLI1 |
| 11 | 129239574 | 129268114 | NFRKB |
| 11 | 133444029 | 133526859 | JAM3 |
| 12 | 891515 | 929124 | RAD52 |
| 12 | 1670507 | 1768105 | ADIPOR2 |
| 12 | 4253198 | 4284777 | CCND2 |
| 12 | 5928300 | 6104097 | VWF |
| 12 | 6179815 | 6217688 | CD9 |
| 12 | 6308184 | 6321522 | TNFRSF1A |
| 12 | 6363617 | 6370993 | LTBR |
| 12 | 6424311 | 6431144 | CD27 |
| 12 | 6513917 | 6517797 | GAPDH |
| 12 | 6549509 | 6586812 | CHD4 |
| 12 | 6727430 | 6732530 | MLF2 |
| 12 | 6751930 | 6757880 | LAG3 |
| 12 | 6768911 | 6800237 | CD4 |
| 12 | 6884212 | 6893666 | LRRC23 |
| 12 | 6893874 | 6903120 | NSE |
| 12 | 6926000 | 6940740 | PTPN6 |
| 12 | 7038277 | 7048594 | C1S |
| 12 | 7057771 | 7136184 | C1R |
| 12 | 7514676 | 7547681 | CD163 |
| 12 | 7773277 | 7793336 | CLEC4C |
| 12 | 8102185 | 8110222 | C3AR1 |
| 12 | 8646028 | 8656706 | AICDA |
| 12 | 9033487 | 9054605 | KLRG1 |
| 12 | 9111570 | 9159825 | A2M |
| 12 | 9796352 | 9804764 | CD69 |
| 12 | 9871343 | 9888868 | KLRF1 |
| 12 | 10036930 | 10043146 | CLEC1B |
| 12 | 10054497 | 10061814 | CLEC12B |
| 12 | 10114348 | 10142872 | CLEC1A |
| 12 | 10160648 | 10174135 | CLEC7A |
| 12 | 10202165 | 10216057 | OLR1 |
| 12 | 10351683 | 10359225 | KLRD1 |
| 12 | 10416219 | 10451632 | KLRK1 |
| 12 | 10489903 | 10498251 | KLRC1 |
| 12 | 10632345 | 10643701 | KLRA1 |
| 12 | 11694054 | 11939590 | ETV6 |
| 12 | 12761575 | 12766569 | CDKN1B |
| 12 | 13019067 | 13044488 | HEBP1 |
| 12 | 14814921 | 14815332 | HIST4H4 |
| 12 | 14986216 | 15005829 | ARHGDIB |
| 12 | 15366753 | 15641602 | PTPRO |
| 12 | 16391342 | 16408610 | MGST1 |
| 12 | 22245201 | 22378915 | ST8SIA1 |
| 12 | 25249446 | 25295121 | KRAS |
| 12 | 26379553 | 26877398 | ITPR2 |
| 12 | 28002284 | 28016183 | PTHLH |
| 12 | 42439046 | 42468164 | IRAK4 |
| 12 | 46417293 | 46438822 | RAPGEF3 |
| 12 | 46462774 | 46479190 | HDAC7 |
| 12 | 46521588 | 46585081 | VDR |
| 12 | 46653015 | 46684552 | COL2A1 |
| 12 | 47247734 | 47250096 | LALBA |
| 12 | 47373018 | 47397048 | CCNT1 |
| 12 | 47658502 | 47662746 | WNT1 |
| 12 | 50723762 | 50739552 | NR4A1 |
| 12 | 51354786 | 51360458 | KRT1 |
| 12 | 51577237 | 51585127 | KRT8 |
| 12 | 51871374 | 51887267 | ITGB7 |
| 12 | 51890620 | 51912303 | RARG |
| 12 | 52121699 | 52126694 | PRR13 |
| 12 | 52132162 | 52159736 | PCBP2 |
| 12 | 53075313 | 53099317 | ITGA5 |
| 12 | 54364622 | 54387894 | ITGA7 |
| 12 | 54405497 | 54409177 | CD63 |
| 12 | 54611212 | 54634072 | DGKA |
| 12 | 54634156 | 54646093 | SILV |
| 12 | 54646825 | 54652834 | CDK2 |
| 12 | 55018929 | 55020460 | IL23A |
| 12 | 55021650 | 55040176 | STAT2 |
| 12 | 55775461 | 55791428 | STAT6 |
| 12 | 55808548 | 55893392 | LRP1 |
| 12 | 56196639 | 56200567 | DDIT3 |
| 12 | 56210360 | 56227245 | DCTN2 |
| 12 | 56305817 | 56313252 | B4GALNT1 |
| 12 | 56442384 | 56447243 | CYP27B1 |
| 12 | 64869283 | 64928652 | IRAK3 |
| 12 | 66834816 | 66839788 | IFNG |
| 12 | 67488246 | 67520481 | MDM2 |
| 12 | 68028430 | 68034280 | LYZ |
| 12 | 78509877 | 78608921 | PAWR |
| 12 | 87410699 | 87498369 | KITLG |
| 12 | 90063166 | 90100937 | DCN |
| 12 | 92326218 | 92360157 | UBE2N |
| 12 | 92487728 | 92494109 | SOCS2 |
| 12 | 97563208 | 97653335 | APAF1 |
| 12 | 99391809 | 99481774 | NR1H4 |
| 12 | 101313806 | 101398454 | IGF1 |
| 12 | 102505198 | 102684630 | STAB2 |
| 12 | 102848318 | 102865833 | HSP90B1 |
| 12 | 107209476 | 107257216 | CMKLR1 |
| 12 | 107539810 | 107551799 | SELPLG |
| 12 | 107563017 | 107649424 | CORO1C |
| 12 | 108019797 | 108033181 | UNG |
| 12 | 110328134 | 110373809 | SH2B3 |
| 12 | 110566278 | 110608122 | IMP |
| 12 | 111340918 | 111432099 | PTPN11 |
| 12 | 111829121 | 111842094 | OAS1 |
| 12 | 111980044 | 112020216 | DTX1 |
| 12 | 116135361 | 116283965 | NOS1 |
| 12 | 119132639 | 119187892 | PXN |
| 12 | 119900931 | 119924697 | HNF1A |
| 12 | 120055060 | 120108239 | P2RX7 |
| 12 | 120548857 | 120564321 | ORAI1 |
| 12 | 121222530 | 121224699 | IL31 |
| 12 | 122762817 | 122810391 | ATP6V0A2 |
| 12 | 123374914 | 123586102 | NCOR2 |
| 12 | 123828126 | 123914472 | SCARB1 |
| 12 | 132217286 | 132246120 | ZNF10 |
| 13 | 19659605 | 19665114 | GJB2 |
| 13 | 20175481 | 20195236 | IL17D |
| 13 | 23893074 | 23984948 | PARP4 |
| 13 | 24844208 | 25493419 | ATP |
| 13 | 27475410 | 27572729 | FLT3 |
| 13 | 27773790 | 27967232 | FLT1 |
| 13 | 29930880 | 29938081 | HMGB1 |
| 13 | 30207668 | 30236556 | ALOX5AP |
| 13 | 32488570 | 32538279 | KL |
| 13 | 35904632 | 35915008 | CCNA1 |
| 13 | 40404164 | 40454418 | ELF1 |
| 13 | 42043794 | 42080148 | TNFSF11 |
| 13 | 44809303 | 44813297 | TPT1 |
| 13 | 45525322 | 45577212 | CPB2 |
| 13 | 45598059 | 45654395 | LCP1 |
| 13 | 46305513 | 46368176 | HTR2A |
| 13 | 47775883 | 47954025 | RB1 |
| 13 | 48178951 | 48181499 | CYSLTR2 |
| 13 | 77367616 | 77447665 | EDNRB |
| 13 | 93889841 | 93929924 | DCT |
| 13 | 94470090 | 94751684 | ABCC4 |
| 13 | 96884476 | 96918245 | RAP2A |
| 13 | 105940098 | 105985338 | EFNB2 |
| 13 | 107657793 | 107665883 | LIG4 |
| 13 | 107719977 | 107757365 | TNFSF13B |
| 13 | 109204184 | 109236915 | IRS2 |
| 13 | 109599311 | 109757459 | COL4A1 |
| 13 | 112808105 | 112822995 | F7 |
| 13 | 112825113 | 112851842 | F10 |
| 13 | 112911086 | 112967392 | CUL4A |
| 13 | 112999469 | 113025742 | LAMP1 |
| 13 | 113546912 | 113590396 | GAS6 |
| 14 | 20007404 | 20015038 | NP |
| 14 | 20319049 | 20320464 | RNASE6 |
| 14 | 20339355 | 20340876 | RNASE1 |
| 14 | 20493469 | 20494434 | RNASE2 |
| 14 | 22375632 | 22386642 | MMP14 |
| 14 | 22656354 | 22658314 | CEBPE |
| 14 | 22895451 | 22904682 | EFS |
| 14 | 22911857 | 22915445 | IL25 |
| 14 | 22921038 | 22947324 | MYH6 |
| 14 | 23675217 | 23678015 | PSME1 |
| 14 | 23682414 | 23685695 | PSME2 |
| 14 | 23700261 | 23705612 | IRF9 |
| 14 | 23788161 | 23802256 | LI |
| 14 | 23852356 | 23855990 | LTB4R |
| 14 | 23907093 | 23918648 | NFATC4 |
| 14 | 24044551 | 24047311 | CMA1 |
| 14 | 24112564 | 24115306 | CTSG |
| 14 | 24170003 | 24173272 | GZMB |
| 14 | 29115439 | 29466650 | PRKD1 |
| 14 | 34940467 | 34943695 | NFKBIA |
| 14 | 49429588 | 49431484 | ARF6 |
| 14 | 51804180 | 51813191 | PTGDR |
| 14 | 51850862 | 51865070 | PTGER2 |
| 14 | 53486206 | 53493362 | BMP4 |
| 14 | 53963396 | 53977898 | CNIH |
| 14 | 54665624 | 54681900 | LGALS3 |
| 14 | 61231991 | 61284729 | HIF1A |
| 14 | 63763505 | 63875021 | ESR2 |
| 14 | 64475624 | 64479284 | GPX2 |
| 14 | 68324127 | 68329538 | ZFP36L1 |
| 14 | 72672931 | 72756862 | PSEN1 |
| 14 | 74478292 | 74492044 | PGF |
| 14 | 74815283 | 74818663 | FOS |
| 14 | 75058536 | 75083085 | BATF |
| 14 | 76857106 | 76867692 | GSTZ1 |
| 14 | 80491621 | 80682399 | TSHR |
| 14 | 87541248 | 87548167 | GPR65 |
| 14 | 90768629 | 90789977 | GPR68 |
| 14 | 93914450 | 93926782 | SERPINA1 |
| 14 | 94148466 | 94160142 | SERPINA3 |
| 14 | 94622318 | 94693512 | DICER1 |
| 14 | 95246057 | 95250201 | TCL1A |
| 14 | 95740949 | 95780536 | BDKRB2 |
| 14 | 95792311 | 95800851 | BDKRB1 |
| 14 | 98705379 | 98807575 | BCL11B |
| 14 | 99774854 | 99814557 | YY1 |
| 14 | 100262981 | 100271223 | DLK1 |
| 14 | 101617138 | 101675776 | HSP90AA1 |
| 14 | 102313568 | 102442381 | TRAF3 |
| 14 | 104290514 | 104297040 | SIVA1 |
| 14 | 104306733 | 104333125 | AKT1 |
| 14 | 104586782 | 104602799 | GPR132 |
| 14 | 104679121 | 104706206 | JAG2 |
| 15 | 29406374 | 29457393 | KLF13 |
| 15 | 30110017 | 30248525 | CHRNA7 |
| 15 | 30797496 | 30814158 | GREM1 |
| 15 | 32097719 | 32144579 | CHRM5 |
| 15 | 36567597 | 36644224 | RASGRP1 |
| 15 | 37660571 | 37676959 | THBS1 |
| 15 | 38367391 | 38387466 | PLCB2 |
| 15 | 39008838 | 39018529 | DLL4 |
| 15 | 39583131 | 39593369 | LTK |
| 15 | 39638523 | 39658818 | TYRO3 |
| 15 | 40492313 | 40537022 | ZFP106 |
| 15 | 41486698 | 41590028 | TP53BP1 |
| 15 | 41825881 | 41852095 | PDIA3 |
| 15 | 42790976 | 42797648 | B2M |
| 15 | 43172143 | 43193651 | DUOX2 |
| 15 | 43666708 | 43689200 | PLDN |
| 15 | 47502750 | 47566814 | FGF7 |
| 15 | 48321438 | 48345218 | HDC |
| 15 | 48356681 | 48434687 | GABPB2 |
| 15 | 49288963 | 49418086 | CYP19A1 |
| 15 | 50386770 | 50608539 | MYO5A |
| 15 | 50836644 | 50869501 | ONECUT1 |
| 15 | 53283093 | 53369293 | RAB27A |
| 15 | 54998124 | 55368004 | TCF12 |
| 15 | 56217770 | 56265401 | AQP9 |
| 15 | 56675801 | 56829469 | ADAM10 |
| 15 | 57184611 | 57204535 | CCNB2 |
| 15 | 58426643 | 58477477 | ANXA2 |
| 15 | 58576755 | 59308794 | RORA |
| 15 | 62235067 | 62242407 | PPIB |
| 15 | 63525051 | 63597088 | DPP8 |
| 15 | 64466678 | 64570935 | MAP2K1 |
| 15 | 64781687 | 64861379 | SMAD6 |
| 15 | 65145248 | 65274586 | SMAD3 |
| 15 | 65622074 | 65886505 | MAP2K5 |
| 15 | 68733948 | 68842904 | UACA |
| 15 | 71763674 | 71793912 | CD276 |
| 15 | 72074066 | 72127206 | PML |
| 15 | 72489375 | 72513329 | SEMA7A |
| 15 | 72828236 | 72835994 | CYP1A2 |
| 15 | 72861767 | 72882557 | CSK |
| 15 | 72924249 | 72952723 | SCAMP2 |
| 15 | 75074608 | 75116726 | PSTPIP1 |
| 15 | 78040289 | 78050698 | BCL2A1 |
| 15 | 79262254 | 79392156 | IL16 |
| 15 | 83724874 | 84093590 | AKAP13 |
| 15 | 86983042 | 86999883 | ISG20 |
| 15 | 87242919 | 87257614 | MFGE8 |
| 15 | 87432428 | 87546591 | ABHD2 |
| 15 | 88129129 | 88150938 | ANPEP |
| 15 | 89061582 | 89159688 | BLM |
| 15 | 89228712 | 89240008 | FES |
| 15 | 97010283 | 97325281 | IGF1R |
| 15 | 99628737 | 99635223 | SELS |
| 16 | 67017 | 75841 | MPG |
| 16 | 751133 | 758866 | MSLN |
| 16 | 1218337 | 1220186 | TPSB2 |
| 16 | 1230678 | 1232555 | TPSAB1 |
| 16 | 2037990 | 2078713 | TSC2 |
| 16 | 2527970 | 2593189 | PDPK1 |
| 16 | 3055313 | 3059668 | IL32 |
| 16 | 3232028 | 3246628 | MEFV |
| 16 | 3642940 | 3648096 | DNASE1 |
| 16 | 3715056 | 3870122 | CREBBP |
| 16 | 4415882 | 4446774 | DNAJA3 |
| 16 | 4466446 | 4500348 | HMOX2 |
| 16 | 9762922 | 10184112 | GRIN2A |
| 16 | 10878557 | 10926340 | CIITA |
| 16 | 11255774 | 11257540 | SOCS1 |
| 16 | 11966464 | 11969425 | TNFRSF17 |
| 16 | 14673907 | 14696027 | PLA2G10 |
| 16 | 15704494 | 15858388 | MYH11 |
| 16 | 15950934 | 16144431 | ABCC1 |
| 16 | 18701778 | 18709157 | RPS15A |
| 16 | 20251874 | 20271538 | UMOD |
| 16 | 21560106 | 21571473 | IGSF6 |
| 16 | 23754822 | 24139063 | PRKCB |
| 16 | 27232751 | 27283599 | IL4R |
| 16 | 27321223 | 27369616 | IL21R |
| 16 | 28418184 | 28425656 | IL27 |
| 16 | 28456162 | 28457996 | NUPR1 |
| 16 | 28850760 | 28858162 | CD19 |
| 16 | 28869818 | 28885266 | NFATC2IP |
| 16 | 28903647 | 28909597 | LAT |
| 16 | 29581800 | 29589322 | SPN |
| 16 | 29739287 | 29766842 | MVP |
| 16 | 29892722 | 29911082 | TAOK2 |
| 16 | 30032926 | 30042131 | MAPK3 |
| 16 | 30102426 | 30107897 | CORO1A |
| 16 | 30391571 | 30442006 | ITGAL |
| 16 | 30815428 | 30822381 | CTF1 |
| 16 | 30952403 | 30958985 | STX4 |
| 16 | 31036487 | 31050206 | MYST1 |
| 16 | 31098953 | 31110598 | FUS |
| 16 | 31120309 | 31121752 | PYCARD |
| 16 | 31134783 | 31135896 | PYDC1 |
| 16 | 31178788 | 31251712 | ITGAM |
| 16 | 31274009 | 31301819 | ITGAX |
| 16 | 31312133 | 31345327 | ITGAD |
| 16 | 49288550 | 49324488 | NOD2 |
| 16 | 49333461 | 49393347 | CYLD |
| 16 | 52025900 | 52083060 | RBL2 |
| 16 | 52082693 | 52094671 | AKTIP |
| 16 | 54070588 | 54098103 | MMP2 |
| 16 | 54394266 | 54424576 | CES1 |
| 16 | 54783648 | 54948648 | GNAO1 |
| 16 | 55950218 | 55957600 | CCL22 |
| 16 | 55963914 | 55976455 | CX3CL1 |
| 16 | 55996179 | 56007475 | CCL17 |
| 16 | 65143966 | 65170537 | CKLF |
| 16 | 65620550 | 65692457 | CBFB |
| 16 | 66073974 | 66075217 | AGRP |
| 16 | 66525907 | 66528254 | PSMB10 |
| 16 | 66531287 | 66535516 | LCAT |
| 16 | 66676875 | 66818338 | NFATC3 |
| 16 | 67131161 | 67290442 | CDH3 |
| 16 | 67328695 | 67426945 | CDH1 |
| 16 | 68156497 | 68296054 | NFAT5 |
| 16 | 68300805 | 68318034 | NQO1 |
| 16 | 70117561 | 70129914 | CHST4 |
| 16 | 70646008 | 70652458 | HP |
| 16 | 70654625 | 70668645 | HPR |
| 16 | 73820429 | 73843004 | BCAR1 |
| 16 | 78185731 | 78192112 | MAF |
| 16 | 80370430 | 80549399 | PLCG2 |
| 16 | 83156706 | 83209170 | COTL1 |
| 16 | 84490274 | 84513710 | IRF8 |
| 16 | 87047514 | 87129074 | ZFPM1 |
| 16 | 87232501 | 87234382 | IL17C |
| 16 | 87237198 | 87244958 | CYBA |
| 16 | 87403379 | 87405843 | AMP |
| 16 | 87403379 | 87405843 | APRT |
| 16 | 87468767 | 87570902 | CBFA2T3 |
| 16 | 88331459 | 88410566 | FANCA |
| 17 | 1272209 | 1306294 | CRK |
| 17 | 1612008 | 1627617 | SERPINF1 |
| 17 | 2234104 | 2251008 | MNT |
| 17 | 2443685 | 2535638 | PAFAH1B1 |
| 17 | 3415490 | 3459454 | TRPV1 |
| 17 | 3564671 | 3651286 | ITGAE |
| 17 | 4480969 | 4491709 | ALOX15 |
| 17 | 4560537 | 4571543 | ARRB2 |
| 17 | 4583580 | 4589863 | CXCL16 |
| 17 | 4657391 | 4673694 | PLD2 |
| 17 | 4683350 | 4742134 | MINK1 |
| 17 | 4776371 | 4779066 | GP1BA |
| 17 | 4789693 | 4792570 | PFN1 |
| 17 | 4841999 | 4868720 | KIF1C |
| 17 | 5276822 | 5283195 | C1QBP |
| 17 | 5345443 | 5428556 | NLRP1 |
| 17 | 6840127 | 6854776 | ALOX12 |
| 17 | 6945364 | 6958803 | ASGR2 |
| 17 | 7393098 | 7401930 | TNFSF12 |
| 17 | 7393098 | 7405649 | TNFSF13 |
| 17 | 7512444 | 7531642 | TP53 |
| 17 | 7549244 | 7555416 | EFNB3 |
| 17 | 8724137 | 8756559 | PIK3R5 |
| 17 | 8865583 | 9084078 | NTN1 |
| 17 | 11864859 | 11987775 | MAP2K4 |
| 17 | 15788955 | 15819935 | ADORA2B |
| 17 | 16395434 | 16413189 | ZNF287 |
| 17 | 16783123 | 16816127 | TNFRSF13B |
| 17 | 17656110 | 17681050 | SREBF1 |
| 17 | 18864714 | 18891061 | GRAP |
| 17 | 21128560 | 21159142 | MAP2K3 |
| 17 | 22823162 | 22974844 | KSR1 |
| 17 | 22982300 | 23000712 | LGALS9 |
| 17 | 23107919 | 23244536 | NOS2 |
| 17 | 23715416 | 23721500 | VTN |
| 17 | 23875085 | 23889300 | FOXN1 |
| 17 | 23897851 | 23903770 | UNC119 |
| 17 | 26446120 | 26728820 | NF1 |
| 17 | 29606408 | 29608331 | CCL2 |
| 17 | 29621352 | 29623368 | CCL7 |
| 17 | 29636799 | 29639312 | CCL11 |
| 17 | 29670178 | 29672532 | CCL8 |
| 17 | 29707583 | 29709741 | CCL13 |
| 17 | 29711511 | 29714365 | CCL1 |
| 17 | 30331650 | 30356201 | LIG3 |
| 17 | 31222609 | 31231490 | CCL5 |
| 17 | 31327648 | 31332636 | CCL16 |
| 17 | 31334804 | 31353125 | CCL14 |
| 17 | 31334804 | 31353125 | CCL15 |
| 17 | 31364209 | 31369118 | CCL23 |
| 17 | 31415755 | 31422953 | CCL18 |
| 17 | 31439715 | 31441619 | CCL3 |
| 17 | 31455332 | 31457127 | CCL4 |
| 17 | 31546383 | 31649843 | CCL3L1 |
| 17 | 31546383 | 31649834 | CCL3L3 |
| 17 | 33939785 | 34015709 | SNIP |
| 17 | 34143675 | 34158084 | PCGF2 |
| 17 | 35097918 | 35138440 | ERBB2 |
| 17 | 35174726 | 35273967 | IKZF3 |
| 17 | 35425213 | 35427591 | CSF3 |
| 17 | 35472588 | 35503644 | THRA |
| 17 | 35718971 | 35767420 | RARA |
| 17 | 35798323 | 35827695 | TOP2A |
| 17 | 35963549 | 35975250 | CCR7 |
| 17 | 36227895 | 36232373 | KRT10 |
| 17 | 37122138 | 37125746 | GAST |
| 17 | 37372284 | 37383280 | CNP |
| 17 | 37604721 | 37681950 | STAT5B |
| 17 | 37693090 | 37717484 | STAT5A |
| 17 | 37718868 | 37794039 | STAT3 |
| 17 | 37941476 | 37949990 | NAGLU |
| 17 | 38084961 | 38087371 | CCR10 |
| 17 | 38215677 | 38229807 | BECN1 |
| 17 | 38256726 | 38263664 | AOC3 |
| 17 | 38306340 | 38318912 | G6PC |
| 17 | 38449839 | 38530994 | BRCA1 |
| 17 | 38576023 | 38719232 | NBR1 |
| 17 | 39509646 | 39556540 | HDAC5 |
| 17 | 39778016 | 39785996 | GRN |
| 17 | 39805075 | 39822399 | ITGA2B |
| 17 | 40338518 | 40348394 | GFAP |
| 17 | 40696270 | 40750197 | MAP3K14 |
| 17 | 41217448 | 41268973 | CRHR1 |
| 17 | 42686206 | 42745075 | ITGB3 |
| 17 | 43165608 | 43178484 | TBX21 |
| 17 | 43328514 | 43361322 | SP2 |
| 17 | 43480744 | 43493840 | NFE2L1 |
| 17 | 43565800 | 43862593 | SKAP1 |
| 17 | 43981231 | 44006809 | HOXB3 |
| 17 | 44007868 | 44010742 | HOXB4 |
| 17 | 44039600 | 44043382 | HOXB7 |
| 17 | 44157124 | 44161110 | HOXB13 |
| 17 | 44927665 | 44947360 | NGFR |
| 17 | 45270669 | 45280378 | TAC4 |
| 17 | 45488729 | 45522843 | ITGA3 |
| 17 | 45616457 | 45633999 | COL1A1 |
| 17 | 50697319 | 50757425 | HLF |
| 17 | 52026273 | 52027542 | NOG |
| 17 | 53625087 | 53636782 | EPX |
| 17 | 53702215 | 53713295 | MPO |
| 17 | 56032335 | 56096818 | PPM1D |
| 17 | 59303104 | 59304821 | CSH2 |
| 17 | 59326005 | 59327719 | CSH1 |
| 17 | 59348295 | 59349930 | GH1 |
| 17 | 59359830 | 59363436 | CD79B |
| 17 | 59433687 | 59437839 | ICAM2 |
| 17 | 59753595 | 59817743 | PECAM1 |
| 17 | 60437295 | 60483216 | GNA13 |
| 17 | 61638613 | 61655992 | APOH |
| 17 | 61729387 | 62237324 | PRKCA |
| 17 | 64922432 | 65050057 | MAP2K6 |
| 17 | 69974116 | 69992526 | CD300A |
| 17 | 70349762 | 70367602 | GRIN2C |
| 17 | 70713191 | 70743449 | NUP85 |
| 17 | 70825752 | 70913384 | GRB2 |
| 17 | 71334901 | 71352393 | UNC13D |
| 17 | 71644011 | 71648161 | FOXJ1 |
| 17 | 71892296 | 71895536 | SPHK1 |
| 17 | 72220508 | 72234476 | JMJD6 |
| 17 | 72241791 | 72245007 | SFRS2 |
| 17 | 73721871 | 73733310 | BIRC5 |
| 17 | 73864458 | 73867753 | SOCS3 |
| 17 | 74360657 | 74433067 | TIMP2 |
| 17 | 76055227 | 76064999 | NPTX1 |
| 17 | 77866034 | 77868769 | CD7 |
| 17 | 77872189 | 77885210 | SECTM1 |
| 18 | 309355 | 490685 | COLEC12 |
| 18 | 895386 | 901050 | ADCYAP1 |
| 18 | 9465529 | 9528105 | RALBP1 |
| 18 | 12244369 | 12267592 | CIDEA |
| 18 | 12775480 | 12874334 | PTPN2 |
| 18 | 13872042 | 13905535 | MC2R |
| 18 | 16783702 | 16945810 | ROCK1 |
| 18 | 19337459 | 19420468 | NPC1 |
| 18 | 19523559 | 19789025 | LAMA3 |
| 18 | 20294590 | 20313918 | HRH4 |
| 18 | 26963213 | 26996817 | DSC1 |
| 18 | 27281729 | 27312663 | DSG3 |
| 18 | 28023985 | 28054362 | MEP1B |
| 18 | 43613464 | 43711510 | SMAD2 |
| 18 | 44700221 | 44731079 | SMAD7 |
| 18 | 45342424 | 45373274 | LIPG |
| 18 | 49934572 | 50005156 | MBD2 |
| 18 | 51040559 | 51406858 | TCF4 |
| 18 | 53418893 | 53440011 | NARS |
| 18 | 54299463 | 54398381 | ALPK2 |
| 18 | 54489597 | 54568350 | MALT1 |
| 18 | 55718216 | 55722517 | PMAIP1 |
| 18 | 56189543 | 56190981 | MC4R |
| 18 | 58143527 | 58204482 | TNFRSF11A |
| 18 | 58941558 | 59137593 | BCL2 |
| 18 | 59473411 | 59480094 | SERPINB3 |
| 18 | 59705921 | 59722100 | SERPINB2 |
| 18 | 65681174 | 65775140 | CD226 |
| 18 | 66107116 | 66148414 | SOCS6 |
| 18 | 72819777 | 72973762 | MBP |
| 18 | 75256759 | 75390310 | NFATC1 |
| 19 | 447489 | 456342 | MADCAM1 |
| 19 | 522324 | 534492 | BSG |
| 19 | 778830 | 783017 | AZU1 |
| 19 | 791984 | 799175 | PRTN3 |
| 19 | 977297 | 990063 | CNN2 |
| 19 | 1054935 | 1057786 | GPX4 |
| 19 | 1058657 | 1125259 | SBNO2 |
| 19 | 1560294 | 1601277 | TCF3 |
| 19 | 2051993 | 2102556 | AP3D1 |
| 19 | 2427134 | 2429257 | GADD45B |
| 19 | 3045407 | 3072452 | GNA11 |
| 19 | 3129765 | 3131329 | S1PR4 |
| 19 | 3545503 | 3557658 | TBXA2R |
| 19 | 4041328 | 4075126 | MAP2K2 |
| 19 | 4180539 | 4188517 | EBI3 |
| 19 | 4275040 | 4289847 | STAP1 |
| 19 | 4275040 | 4289847 | STAP2 |
| 19 | 4608565 | 4621415 | C19ORF10 |
| 19 | 4766991 | 4769451 | TICAM1 |
| 19 | 6161392 | 6230959 | MLLT1 |
| 19 | 6482036 | 6486933 | TNFSF9 |
| 19 | 6536850 | 6542163 | CD70 |
| 19 | 6615565 | 6621599 | TNFSF14 |
| 19 | 6628846 | 6671662 | C3 |
| 19 | 6723721 | 6808371 | VAV1 |
| 19 | 7063265 | 7245011 | INSR |
| 19 | 7659662 | 7672997 | FCER2 |
| 19 | 7710882 | 7718406 | CD209 |
| 19 | 7734080 | 7740490 | CLEC4M |
| 19 | 7874764 | 7885362 | MAP2K7 |
| 19 | 8023933 | 8033546 | CCL25 |
| 19 | 8273010 | 8279239 | CD320 |
| 19 | 8460940 | 8473495 | PRAM1 |
| 19 | 8491998 | 8548307 | MYO1F |
| 19 | 9806998 | 9821358 | PIN1 |
| 19 | 10083196 | 10087062 | P2RY11 |
| 19 | 10105022 | 10166811 | DNMT1 |
| 19 | 10193108 | 10202948 | S1PR2 |
| 19 | 10242778 | 10258291 | ICAM1 |
| 19 | 10261654 | 10268452 | ICAM5 |
| 19 | 10305453 | 10311300 | ICAM3 |
| 19 | 10322208 | 10352211 | TYK2 |
| 19 | 10484622 | 10489112 | S1PR5 |
| 19 | 10538138 | 10540655 | CDKN2D |
| 19 | 10843252 | 10894447 | CARM1 |
| 19 | 10932605 | 11033952 | SMARCA4 |
| 19 | 11061131 | 11105490 | LDLR |
| 19 | 11349474 | 11356019 | EPOR |
| 19 | 12763309 | 12765124 | JUNB |
| 19 | 12768634 | 12773694 | PRDX2 |
| 19 | 12910422 | 12916303 | CALR |
| 19 | 13933352 | 13978097 | RFX1 |
| 19 | 14003261 | 14025025 | IL27RA |
| 19 | 14063508 | 14089559 | PRKACA |
| 19 | 14353212 | 14380533 | CD97 |
| 19 | 14444278 | 14447174 | PTGER1 |
| 19 | 15131443 | 15172792 | NOTCH3 |
| 19 | 15440462 | 15451312 | PGLYRP2 |
| 19 | 15849833 | 15869884 | CYP4F2 |
| 19 | 15884181 | 15906326 | CYP4F11 |
| 19 | 16105837 | 16130375 | HSH2D |
| 19 | 16296650 | 16299337 | KLF2 |
| 19 | 16860825 | 16863828 | F2RL3 |
| 19 | 17073527 | 17185103 | MYO9B |
| 19 | 17374755 | 17377384 | BST2 |
| 19 | 17788321 | 17793320 | INSL3 |
| 19 | 17797960 | 17819800 | JAK3 |
| 19 | 18031370 | 18058697 | IL12RB1 |
| 19 | 18125015 | 18142344 | PIK3R2 |
| 19 | 18145578 | 18149927 | IFI30 |
| 19 | 18251570 | 18253432 | JUND |
| 19 | 18414473 | 18493918 | ELL |
| 19 | 35125264 | 35198451 | C19ORF2 |
| 19 | 38482775 | 38485160 | CEBPA |
| 19 | 38556448 | 38565431 | CEBPG |
| 19 | 39547908 | 39583076 | GPI |
| 19 | 40465249 | 40467883 | HAMP |
| 19 | 40511951 | 40530102 | CD22 |
| 19 | 40895669 | 40899779 | ZBTB32 |
| 19 | 41087145 | 41091026 | TYROBP |
| 19 | 43591537 | 43608785 | RASGRP4 |
| 19 | 43770120 | 43800483 | MAP4K1 |
| 19 | 43830166 | 43913010 | ACTN4 |
| 19 | 44082454 | 44091373 | NFKBIB |
| 19 | 44450996 | 44452572 | IL28A |
| 19 | 44478804 | 44481151 | IL29 |
| 19 | 44589326 | 44591885 | ZFP36 |
| 19 | 44913735 | 44920508 | CLC |
| 19 | 45428063 | 45483105 | AKT2 |
| 19 | 46416662 | 46459510 | AXL |
| 19 | 46528490 | 46551656 | TGFB1 |
| 19 | 46904369 | 46967953 | CEACAM5 |
| 19 | 46951340 | 46967953 | CEACAM6 |
| 19 | 46992373 | 47007431 | CEACAM3 |
| 19 | 47055827 | 47067322 | RPS19 |
| 19 | 47073029 | 47077278 | CD79A |
| 19 | 47079106 | 47103440 | ARHGEF1 |
| 19 | 47284512 | 47328470 | POU2F2 |
| 19 | 47703298 | 47724479 | CEACAM1 |
| 19 | 47776234 | 47790890 | CEACAM8 |
| 19 | 47917634 | 47936508 | PSG3 |
| 19 | 48842087 | 48866342 | PLAUR |
| 19 | 48962526 | 48977249 | KCNN4 |
| 19 | 49839065 | 49858689 | PVR |
| 19 | 49943870 | 49955140 | BCL3 |
| 19 | 50041232 | 50084325 | PVRL2 |
| 19 | 50086333 | 50098775 | TOMM40 |
| 19 | 50100878 | 50104489 | APOE |
| 19 | 50109760 | 50114446 | APOC1 |
| 19 | 50196551 | 50233292 | RELB |
| 19 | 50373890 | 50375003 | BLOC1S3 |
| 19 | 50604711 | 50619017 | ERCC1 |
| 19 | 50663092 | 50670276 | FOSB |
| 19 | 51108314 | 51109876 | NOS2 |
| 19 | 51214280 | 51218163 | PGLYRP1 |
| 19 | 52415924 | 52426291 | BBC3 |
| 19 | 52504943 | 52517167 | C5AR1 |
| 19 | 53310514 | 53365372 | LIG1 |
| 19 | 53814359 | 53825473 | SPHK2 |
| 19 | 54149928 | 54156866 | BAX |
| 19 | 54160377 | 54161947 | FTL |
| 19 | 54530488 | 54535671 | CD37 |
| 19 | 54669297 | 54681299 | FLT3LG |
| 19 | 54708303 | 54721400 | FCGRT |
| 19 | 54854641 | 54860926 | IRF3 |
| 19 | 55084722 | 55124574 | IL4I1 |
| 19 | 55571496 | 55578079 | NR1H2 |
| 19 | 55614027 | 55624058 | SPIB |
| 19 | 55918416 | 55920791 | CLEC11A |
| 19 | 56049982 | 56055832 | KLK3 |
| 19 | 56191075 | 56196770 | KLK8 |
| 19 | 56251275 | 56260179 | KLK13 |
| 19 | 56319976 | 56325378 | SIGLEC9 |
| 19 | 56337369 | 56348595 | SIGLEC7 |
| 19 | 56420146 | 56435086 | CD33 |
| 19 | 56605087 | 56613263 | SIGLEC10 |
| 19 | 56646063 | 56653520 | SIGLEC8 |
| 19 | 56686424 | 56696855 | SIGLEC12 |
| 19 | 56940838 | 56946962 | FPR1 |
| 19 | 59289746 | 59295960 | OSCAR |
| 19 | 59411958 | 59516221 | LILRB3 |
| 19 | 59432280 | 59438536 | LILRA6 |
| 19 | 59446081 | 59452979 | LILRB5 |
| 19 | 59469486 | 59476818 | LILRB2 |
| 19 | 59491668 | 59496050 | LILRA3 |
| 19 | 59557046 | 59568533 | LAIR1 |
| 19 | 59777070 | 59790833 | LILRA2 |
| 19 | 59796924 | 59840816 | LILRB1 |
| 19 | 59796924 | 59804352 | LILRA1 |
| 19 | 59865935 | 59871658 | LILRB4 |
| 19 | 59941785 | 59956315 | KIR2DL3 |
| 19 | 59973076 | 59987590 | KIR2DL1 |
| 19 | 60006878 | 60017784 | KIR2DL4 |
| 19 | 60019704 | 60070474 | KIR3DL1 |
| 19 | 60035985 | 60051835 | KIR2DS4 |
| 19 | 60077360 | 60093651 | FCAR |
| 19 | 60109337 | 60116251 | NCR1 |
| 19 | 60216886 | 60241444 | GP6 |
| 19 | 60354949 | 60360858 | TNNI3 |
| 19 | 60567568 | 60573626 | IL11 |
| 19 | 61379200 | 61388956 | GALP |
| 20 | 86185 | 87804 | DEFB127 |
| 20 | 801298 | 844960 | ANG4 |
| 20 | 1297622 | 1321745 | FKBP1A |
| 20 | 1462898 | 1548689 | SIRPB1 |
| 20 | 1557797 | 1586425 | SIRPG |
| 20 | 1822812 | 1868539 | SIRPA |
| 20 | 2972267 | 2974391 | GNRH2 |
| 20 | 3399675 | 3579760 | ATRN |
| 20 | 3615618 | 3635775 | SIGLEC1 |
| 20 | 3775483 | 3795972 | MAVS |
| 20 | 4149815 | 4177659 | ADRA1D |
| 20 | 4614796 | 4630234 | PRNP |
| 20 | 6696744 | 6708910 | BMP2 |
| 20 | 8061295 | 8813545 | PLCB1 |
| 20 | 10566333 | 10602590 | JAG1 |
| 20 | 21634363 | 21644620 | PAX1 |
| 20 | 22509822 | 22514102 | FOXA2 |
| 20 | 22974270 | 22978301 | THBD |
| 20 | 23007993 | 23014977 | CD93 |
| 20 | 23562293 | 23566574 | CST3 |
| 20 | 24877865 | 24888562 | CST7 |
| 20 | 29354676 | 29360049 | DEFB116 |
| 20 | 29420088 | 29424824 | DEFB118 |
| 20 | 29656752 | 29657974 | ID1 |
| 20 | 29715923 | 29774317 | BCL2L1 |
| 20 | 30103717 | 30153316 | HCK |
| 20 | 30813851 | 30860822 | DNMT3B |
| 20 | 31727149 | 31737854 | E2F1 |
| 20 | 32414744 | 32562858 | ITCH |
| 20 | 32766239 | 32877094 | NCOA6 |
| 20 | 32979897 | 33007262 | GSS |
| 20 | 33223434 | 33228825 | PROCR |
| 20 | 33278116 | 33328218 | MMP24 |
| 20 | 34674335 | 34707972 | SLA2 |
| 20 | 35059592 | 35157824 | RBL1 |
| 20 | 35406501 | 35467233 | SRC |
| 20 | 36190278 | 36227114 | TGM2 |
| 20 | 36365998 | 36399319 | BPI |
| 20 | 36408298 | 36439067 | LBP |
| 20 | 38747932 | 38751290 | MAFB |
| 20 | 39199574 | 39237771 | PLCG1 |
| 20 | 41729122 | 41778536 | MYBL2 |
| 20 | 42417854 | 42493443 | HNF4A |
| 20 | 42681577 | 42713790 | ADA |
| 20 | 43028533 | 43142005 | STK4 |
| 20 | 43185480 | 43186520 | WFDC12 |
| 20 | 43236911 | 43238598 | PI3 |
| 20 | 43314292 | 43316620 | SLPI |
| 20 | 43960803 | 43974193 | PLTP |
| 20 | 44070953 | 44078606 | MMP9 |
| 20 | 44180312 | 44191791 | CD40 |
| 20 | 48240782 | 48242619 | CEBPB |
| 20 | 48560297 | 48634491 | PTPN1 |
| 20 | 49441172 | 49592665 | NFATC2 |
| 20 | 55359551 | 55386925 | RAE1 |
| 20 | 56848189 | 56919644 | GNAS |
| 20 | 57308893 | 57334441 | EDN3 |
| 20 | 60223421 | 60228707 | HRH3 |
| 20 | 60317517 | 60375763 | LAMA5 |
| 20 | 61445108 | 61463192 | CHRNA4 |
| 20 | 61760090 | 61800477 | TNFRSF6B |
| 21 | 26174732 | 26465003 | APP |
| 21 | 31412606 | 31853161 | TIAM1 |
| 21 | 31953805 | 31963112 | SOD1 |
| 21 | 33524100 | 33558688 | IFNAR2 |
| 21 | 33560541 | 33591390 | IL10RB |
| 21 | 33619083 | 33653996 | IFNAR1 |
| 21 | 33697071 | 33731696 | IFNGR2 |
| 21 | 35081968 | 35343465 | RUNX1 |
| 21 | 39099718 | 39118746 | ETS2 |
| 21 | 41655819 | 41702739 | MX2 |
| 21 | 41720023 | 41753008 | MX1 |
| 21 | 42492867 | 42590421 | ABCG1 |
| 21 | 42605232 | 42608775 | TFF3 |
| 21 | 42655461 | 42659713 | TFF1 |
| 21 | 42697087 | 42740841 | UBASH3A |
| 21 | 43267711 | 43326757 | PKNOX1 |
| 21 | 44471151 | 44485262 | ICOSLG |
| 21 | 44530190 | 44542528 | AIRE |
| 21 | 44597911 | 44687392 | TRPM2 |
| 21 | 45130298 | 45165303 | ITGB2 |
| 21 | 45649524 | 45758061 | COL18A1 |
| 21 | 45759056 | 45786779 | SLC19A1 |
| 22 | 16596905 | 16637258 | BID |
| 22 | 19601713 | 19637889 | CRKL |
| 22 | 20443948 | 20551970 | MAPK1 |
| 22 | 20929199 | 20929926 | VPREB1 |
| 22 | 21742668 | 21797221 | GNAZ |
| 22 | 21852551 | 21990223 | BCR |
| 22 | 22245313 | 22252495 | IGLL1 |
| 22 | 22445035 | 22456502 | MMP11 |
| 22 | 22566564 | 22567409 | MIF |
| 22 | 22945638 | 22971028 | GGT5 |
| 22 | 23159091 | 23168324 | ADORA2A |
| 22 | 27413730 | 27483488 | CHEK2 |
| 22 | 27520548 | 27526560 | XBP1 |
| 22 | 27994016 | 28026515 | EWSR1 |
| 22 | 28234156 | 28279736 | THOC5 |
| 22 | 28966442 | 28972748 | LIF |
| 22 | 28988819 | 28992840 | OSM |
| 22 | 29280623 | 29290876 | GAL3ST1 |
| 22 | 30051790 | 30072249 | PATZ1 |
| 22 | 31526801 | 31589027 | TIMP3 |
| 22 | 34107086 | 34120194 | HMOX1 |
| 22 | 34866324 | 34892171 | APOL3 |
| 22 | 34979069 | 34993522 | APOL1 |
| 22 | 35007271 | 35113927 | MYH9 |
| 22 | 35586990 | 35604004 | NCF4 |
| 22 | 35648167 | 35664763 | CSF2RB |
| 22 | 35851827 | 35875908 | IL2RB |
| 22 | 35951257 | 35970251 | RAC2 |
| 22 | 36296199 | 36305970 | LGALS2 |
| 22 | 36401558 | 36405753 | LGALS1 |
| 22 | 36837448 | 36907707 | PLA2G6 |
| 22 | 37431752 | 37459536 | GTPBP1 |
| 22 | 37803081 | 37813693 | APOBEC3G |
| 22 | 38627031 | 38697330 | GRAP2 |
| 22 | 39818552 | 39906024 | EP300 |
| 22 | 40347240 | 40389998 | XRCC6 |
| 22 | 40650981 | 40652728 | TNFRSF13C |
| 22 | 41106357 | 41158345 | NFAM1 |
| 22 | 44925162 | 45018317 | PPARA |
| 22 | 45458971 | 45512816 | CERK |
| 22 | 49293537 | 49308766 | NCAPH2 |
| 22 | 49523512 | 49530592 | ACR |
| X | 1274886 | 1291530 | CRLF2 |
| X | 1347700 | 1388827 | CSF2RA |
| X | 1415508 | 1461581 | IL3RA |
| X | 2619227 | 2669348 | CD99 |
| X | 3532410 | 3641649 | PRKX |
| X | 12795122 | 12818400 | TLR7 |
| X | 12834678 | 12851207 | TLR8 |
| X | 12903147 | 12905266 | TMSB4X |
| X | 15428872 | 15484572 | BMX |
| X | 24621976 | 24925021 | POLA1 |
| X | 31047265 | 33267647 | DMD |
| X | 37524263 | 37557658 | CYBB |
| X | 47326633 | 47331133 | TIMP1 |
| X | 47368571 | 47374648 | CFP |
| X | 48427152 | 48434759 | WAS |
| X | 48529905 | 48537659 | GATA1 |
| X | 48545430 | 48568324 | HDAC6 |
| X | 48655404 | 48661228 | PIM2 |
| X | 48994353 | 49008232 | FOXP3 |
| X | 64804235 | 64878517 | MSN |
| X | 65158304 | 65176610 | VSIG4 |
| X | 66680598 | 66860844 | AR |
| X | 67965555 | 67978727 | EFNB1 |
| X | 69270042 | 69302896 | IGBP1 |
| X | 70243983 | 70248128 | IL2RG |
| X | 70752491 | 70755092 | CXCR3 |
| X | 72583814 | 72591145 | CDX4 |
| X | 77052875 | 77192208 | ATP7A |
| X | 77414786 | 77469743 | CYSLTR1 |
| X | 99984969 | 100015990 | NOX1 |
| X | 100491097 | 100527838 | BTK |
| X | 100539434 | 100549657 | GLA |
| X | 102918409 | 102934201 | PLP1 |
| X | 105823723 | 105926900 | RNF128 |
| X | 106843112 | 106905673 | TSC22D3 |
| X | 113724806 | 114050880 | HTR2C |
| X | 114144795 | 114158463 | IL13RA2 |
| X | 117745586 | 117812523 | IL13RA1 |
| X | 122821728 | 122875502 | XIAP |
| X | 123307830 | 123334685 | SH2D1A |
| X | 123339503 | 123925347 | ODZ1 |
| X | 128607006 | 128616595 | APLN |
| X | 129026585 | 129072147 | ELF4 |
| X | 129091019 | 129127489 | AIFM1 |
| X | 133421922 | 133462362 | HPRT1 |
| X | 135558001 | 135570214 | CD40LG |
| X | 135575376 | 135691169 | ARHGEF6 |
| X | 138440560 | 138473283 | F9 |
| X | 149685467 | 149817837 | CD99L2 |
| X | 149902420 | 149909904 | HMGB3 |
| X | 151633774 | 151673020 | MAGEA2 |
| X | 151685308 | 151688896 | MAGEA3 |
| X | 152413604 | 152428198 | BGN |
| X | 152619146 | 152643081 | BCAP31 |
| X | 152780580 | 152794505 | L1CAM |
| X | 152929150 | 152938536 | IRAK1 |
| X | 153412799 | 153428981 | G6PD |
| X | 153429255 | 153446455 | IKBKG |
| X | 153556720 | 153632542 | GAB3 |
| X | 153943095 | 153952830 | MTCP1 |
| Y | 19611913 | 19614093 | CD24 |
| Y | 20326690 | 20366212 | HYA |
